# Efficacy of intraoperative indocyanine green videoangiography (ICG-VA) and FLOW 800 in the surgical management of intracranial aneurysms: a systematic review and meta-analysis

**DOI:** 10.1007/s00701-026-06779-6

**Published:** 2026-01-26

**Authors:** Albert Gabriel Turpo-Peqqueña, Santiago Alejandro Santos-Vargas, Harlly Romed Loza-Chipa, Francisco Martins Lamas, George Alejandro Espinoza-Laura, Claudia Solange Núñez-Basurco, Diego Napoleon Medina-Neira, Valeria Alejandra Benites-Bustamante, Rayza Ruth Osorio-Pacheco, Josue Rodrigo Turpo-Peqqueña, Gladys Huanca-Quispe, Cristhian Adolfo Vizcarra-Vizcarra, Badhin Gómez, Julian Alejandro Rivillas, Richard Hernández-Mayori

**Affiliations:** 1 Faculty of Human Medicine, Universidad Católica de Santa María, Arequipa, Peru; 2 Faculty of Human Medicine, Universidade Federal de Ciências da Saúde de Porto Alegre (UFCSPA), Porto Alegre, Brasil; 3 Faculty of Human Medicine, Universidad de San Martín de Porres, Arequipa, Perú; 4 Faculty of Nursing, Universidad Nacional de San Agustin, Arequipa, Perú; 5 Faculty of Medicine, Université de Montréal, Montreal, Canada; 6 Center for Molecular Engineering Research (CIIM), Universidad Católica de Santa María, Arequipa, Perú; 7 Neurology Service, Valle del Lili Foundation, Cali, Colombia; 8 Neurosurgery Service, Hospital Regional Honorio Delgado Espinoza (HRHDE), Arequipa, Perú

**Keywords:** Indocyanine green, FLOW 800, Intracranial aneurysm, Intraoperative imaging

## Abstract

**Background:**

Intraoperative assessment of aneurysm clipping remains technically challenging, particularly in identifying misclippings, aneurysmal remnants, and vessel compromise. Indocyanine green videoangiography (ICG-VA) provides real-time visualization but lacks hemodynamic quantification. FLOW 800 is a semi quantitative analysis tool that may assist intraoperative blood flow evaluation. This meta-analysis aims to evaluate intraoperative outcomes reported with the combined use of ICG-VA and FLOW 800 in intracranial aneurysm surgery, focusing on intraoperative outcomes.

**Methods:**

A systematic review was conducted in five databases (PubMed, Embase, Scopus, Web of Science, CENTRAL). The protocol was registered in PROSPERO (CRD420251014600). Twelve studies were included in the qualitative synthesis (384 aneurysms), of which eight contributed quantitative data to the meta-analysis (277 aneurysms). Pooled proportions of misclipping, aneurysmal remnant, vascular stenosis/occlusion, and clip repositioning were calculated using a random-effects model. Subgroup analyses, meta-regression, leave-one-out sensitivity analysis, and assessment of publication bias (funnel plot) were performed. Risk of bias was assessed using the QUADAS-2 tool.

**Results:**

The pooled intraoperative detection rates using ICG-VA and FLOW 800 were: misclipping 9.36% (95% CI: 4.75–17.64), aneurysm remnant 6.55% (95% CI: 3.29–12.65), vessel stenosis or occlusion 6.90% (95% CI: 3.28–13.96), and clip repositioning 8.13% (95% CI: 4.05–15.63). Retrospective studies showed higher detection rates than prospective ones. Meta-regression identified study design as a significant predictor for all outcomes (*p* < 0.0001), while older patient age was associated with increased remnant detection (*p* = 0.0247) and clip repositioning (*p* = 0.0073). Funnel plots revealed slight asymmetry, and GRADE evaluation indicated moderate certainty for misclipping and clip repositioning, and low certainty for remnants and stenosis.

**Conclusions:**

The combined use of ICG-VA and FLOW 800 may serve as a complementary intraoperative tool for the assessment of misclipping, residual aneurysm, and flow disturbances reported during aneurysm surgery. These findings support its role as a complementary intraoperative tool. However, due to limited validation against angiographic standards, it should not replace DSA. Further prospective comparative studies are warranted to validate these findings against angiographic standards and better define its intraoperative role in neurosurgical practice.

**Supplementary Information:**

The online version contains supplementary material available at 10.1007/s00701-026-06779-6.

## Introduction

The primary goal of treating intracranial aneurysms is to prevent rebleeding in ruptured cases [21] and to avoid future rupture in unruptured ones. Therapeutic options include surgical techniques, such as microsurgical clipping, and endovascular techniques, such as coil embolization. The choice of method depends on various risk factors, including family history, aneurysm size, morphology, and location [14]. However, current guidelines recommend individualizing treatment based on the characteristics of both the aneurysm and the patient, with surgical clipping being prioritized in complex cases where endovascular access is not feasible [8, 17].

Microsurgical clipping is a high-risk neurosurgical procedure with several challenges that may compromise its success [22]. One such challenge is the presence of residual aneurysmal remnants, which have been reported in up to 4% of patients on postoperative angiographic control, due to incomplete clipping, thereby maintaining the risk of rupture [30]. Another critical issue is inadvertent clipping of adjacent arterial branches that are not adequately visualized during surgery [31], potentially leading to cerebral ischemia and stroke [39]. As a result, clip repositioning is required in approximately 40.6% of cases [20]. To reduce these risks, surgeons now use intraoperative tools like neurophysiological monitoring, flowmetry, and indocyanine green videoangiography (ICG-VA) to improve safety during clipping, although digital subtraction angiography (DSA) remains the gold standard for intraoperative vascular assessment [8, 17].

Despite its usefulness, direct visual assessment with ICG-VA may miss subtle hemodynamic alterations. To overcome this limitation, FLOW 800 (Zeiss Meditec, Oberkochen, Germany), a software that transforms fluorescence signals into color-coded maps, has been introduced, allowing semi-quantitative intraoperative assessment of cerebral flow dynamics [36]. This tool is particularly valuable in complex anatomical regions such as the anterior communicating artery, where conventional visualization is often limited [3]. Although its use has been described in several observational studies, there is still a lack of quantitative evidence supporting the combined efficacy of FLOW 800 and ICG-VA. Therefore, this meta-analysis aims to evaluate intraoperative findings reported with the combined use of ICG-VA and FLOW 800 in intracranial aneurysm surgery, focusing on intraoperative outcomes such as the rate of misclipping, false negatives, aneurysm remnant detection, and vessel stenosis or occlusion.

## Methods

### Protocol registration

This study was conducted in accordance with the Preferred Reporting Items for Systematic Reviews and Meta-Analyses (PRISMA) guidelines [28] and the Cochrane Handbook for Systematic Reviews of Interventions [16]. The protocol was previously registered in the International Prospective Register of Systematic Reviews (PROSPERO) under the ID: CRD420251014600.

### Search strategy

A systematic and comprehensive search was performed in five electronic databases: PubMed, Embase, Scopus, Web of Science, CENTRAL (Cochrane Library), up to February 20, 2025, with no restrictions on language, publication date, or study type. The search focused on terms related to indocyanine green (ICG), videoangiography techniques including FLOW 800, and intracranial aneurysm treatments such as clipping, surgery, or endovascular procedures. The detailed search strategies for each database are available in the Supplementary Material (Supplemental Table 1).

### Inclusion and exclusion criteria

Inclusion criteria:

- Randomized or nonrandomized observational studies.
- Studies including at least five patients undergoing surgical clipping of intracranial aneurysms.
- Studies reporting the intraoperative use of both indocyanine green videoangiography (ICG-VA) and FLOW 800 software, regardless of which specific outcomes were reported.
- No exclusion was made based on the type of surgical technique (i.e., clipping alone or clipping combined with bypass/anastomosis).
- Eligible outcomes included aneurysm remnant, vessel stenosis/occlusion, misclipping, etc.

Exclusion criteria:

- Case reports, systematic reviews, meta-analyses, narrative reviews, clinical guidelines, editorials, letters, book chapters, basic science (in vivo or in vitro), and conference abstracts.
- Studies that did not use both ICG-VA and FLOW 800 concurrently.
- Studies focusing on using fluorescein videoangiography.
- Studies with fewer than five patients.

### Study selection

Five reviewers independently screened titles and abstracts. Full-text articles of potentially eligible studies were then assessed based on the predefined inclusion criteria. Any discrepancies were resolved by consensus or, when necessary, through consultation with the senior author.

### Data extraction and outcomes assessed

Five reviewers independently extracted relevant data using a standardized form. Extracted variables included study characteristics (authors, year, country, study design, sample size), clinical features (patient age, sex, aneurysm type and location), procedural details, and outcomes (clip repositioning, false negatives and residual aneurysms). All outcomes were extracted and analyzed per aneurysm. Denominators were harmonized consistently across the manuscript to avoid misinterpretation.

### Verification of intraoperative findings

In the data extraction section, it was also recorded whether each study verified the ICG-VA + FLOW 800 findings using an independent reference standard, such as digital subtraction angiography (DSA), computed tomography angiography (CTA), or intraoperative puncture; and whether such verification altered the intraoperative impression (false positives or false negatives). The verification practices of the studies are summarized in Supplementary Table 2.

### Outcomes and definitions

All intraoperative outcomes were assessed using the combined use of ICG-VA and FLOW 800. Operational definitions were standardized as follows (Supplemental Table 3):

- Misclipping: Presence of either an aneurysm remnant or vessel stenosis/occlusion.
- Aneurysm remnant: Persistent filling of the aneurysm sac or neck after clip application.
- Vessel stenosis/occlusion: Unintended reduction or absence of arterial flow in a parent or branching vessel adjacent to the clip.
- Clip repositioning Any intraoperative adjustment of clip position performed in response to abnormal perfusion patterns detected during surgery. It was analyzed separately, as not all repositionings indicate misclipping or error.

When remnant and stenosis occurred within the same aneurysm, the case was counted once under the composite outcome “misclipping” to avoid double counting. When they occurred in different aneurysms, each was counted as an independent misclipping event. Examples of each operational definition are provided in Supplemental Table 3.

### Quality assessment

The methodological quality and risk of bias of the included studies were evaluated using the QUADAS-2 tool [38], which is specifically designed for diagnostic accuracy studies. Given that the outcomes in this review represent intraoperative detection yields rather than therapeutic comparisons, QUADAS-2 was considered the most appropriate framework. However, because independent reference standards (e.g., intraoperative DSA) were limited, signaling questions were adapted to account for partial or selective validation. ROBINS-I was considered, but its focus on comparative effectiveness studies made it less suitable for this diagnostic accuracy context.

### Statistical analysis

All statistical analyses were conducted using R statistical software (version 4.2.3). A single-arm meta-analysis was performed with a random-effects model. Categorical outcomes were summarized as pooled proportions with corresponding 95% confidence intervals (CI). Between-study heterogeneity was assessed with the Cochrane Q test (*p* < 0.05), the I^2^ statistic (low, moderate, high: 25%, 50%, 75%), and τ^2^ to quantify variance [28]. Publication bias was explored through visual inspection of funnel plots; however, given that fewer than 10 studies contributed to each endpoint, any asymmetry is difficult to interpret and small-study bias analyses should be considered exploratory only. Finally, a leave-one-out sensitivity analysis was conducted to evaluate the influence of individual studies on the pooled estimates. A pooled proportion represents the overall combined rate of an outcome across all included studies, weighted by sample size.

### Meta-regression

A mixed-effects model was applied using the transformed proportion of each outcome as the dependent variable [15]. The continuous covariates included year of publication, mean patient age, and follow-up duration; the categorical covariates included surgical technique, laterality, and irrigated artery, with their respective reference categories. The random-effects model enabled the identification of significant predictors and quantified the extent to which between-study heterogeneity was attributable to these factors.

### Certainty of evidence

The overall certainty of evidence for the main outcomes was assessed using the GRADE (Grading of Recommendations Assessment, Development and Evaluation) approach, taking into account the risk of bias, inconsistency, indirectness, imprecision, and publication bias [13].

## Results

### Study characteristics

**Fig. 1:**
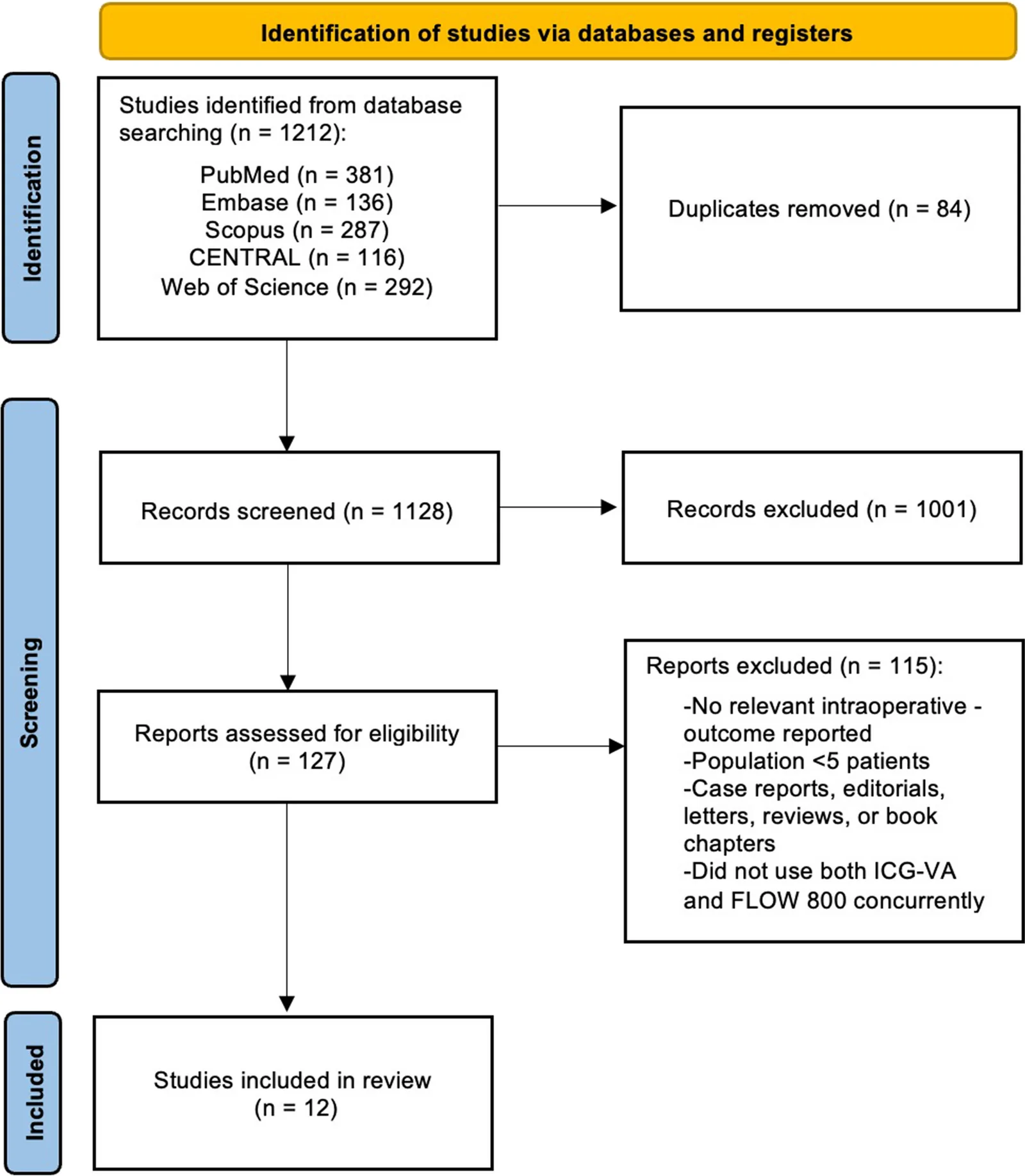
PRISMA flow diagram. PRISMA, preferred reporting items for systematic reviews and meta-analysis. ICG-VA: Indocyanine Green Videoangiography

The initial literature search identified a total of 1212 records, which was reduced to 1128 after removing 84 duplicates. After screening titles and abstracts, 1001 records were excluded. A total of 127 full-text articles were assessed for eligibility. Of these, 115 were excluded due to one or more of the following reasons: lack of relevant intraoperative outcomes, population size fewer than five patients, or failure to use both ICG-VA and FLOW 800 concurrently. Finally, twelve clinical studies met the inclusion criteria and were included in the systematic review and meta-analysis [3, 10, 18, 19, 23–25, 27, 32, 36, 41, 43]. The complete workflow of the study selection process is illustrated in Fig. 1.

Twelve clinical studies published between 2011 and 2024 were included in this meta-analysis, all evaluating the intraoperative use of ICG-VA combined with FLOW 800 for the microsurgical treatment of intracranial aneurysms. A total of 335 patients and 384 aneurysms were analyzed, of which 277 aneurysms contributed quantitative outcome data to the meta-analysis. Nine studies had a retrospective design [3, 10, 18, 23, 25, 27, 32, 36, 41], while three were prospective [19, 24, 43]. No randomized controlled trials were identified. Most aneurysms were located in the anterior circulation, with the middle cerebral artery (MCA), anterior communicating artery (AComA), and internal carotid artery (ICA). Aneurysm sizes ranged from 4.5 mm to 16.8 mm. Patient age ranged from 42 to 69.3 years. The ICG bolus doses varied between 0.25 mg and 25 mg per injection, either as a fixed dose or weight-based. Detailed baseline characteristics of the included studies are presented in Table 1.

**Table 1:** Baseline characteristics of the included studies’ populations

Author, Year	Country	Study design	Patients with Aneurysm	Aneurysms	Ruptured/Unruptured	Location	Mean Size (mm)	Mean Age (Years)	Sex (M/F)	ICG dose
Oda, 2011 [27]	Japan	Retrospective	39	43	7/36	ICA, MCA, AComA, AChoA	NR	61.1	12/27	25 mg (dissolved in 5 ml water or 0.25 mg/kg)
Ye, 2013 [43]	China	Prospective	45	45	12/33	NR	NR	42 ± NR	25/20	25 mg
Murai, 2016 [24]	Japan	Prospective	13	13	NR	MCA	6–11 mm	63.7 ± NR	5/8	0.3–0.5 mg/kg
Goertz, 2019 [10]	Germany	Retrospective	54	60	47/13	ACA, MCA, ACP, ICA	5.7 ± 2.8	53.6 ± 11.6	15/39	10 mg
Shah, 2019 [36]	United States	Retrospective	11	11	NR	Anterior, posterior circulation	8.0 ± NR	52 ± NR	9/14	25 mg
Chavan, 2020 [3]	Japan	Retrospective	10	10	0/10	AComA	4.5 ± 1.7	69.3 ± NR	4/6	0.25 mg/kg
Xue, 2021 [41]	China	Retrospective	32	42	NR	ICA, PComA, AComA, BA, PCA, AICA, PICA, SCA	16.8 ± 16.3	53.9 ± 14.5	5/27	NR
Khasanov, 2023 [18]	Japan	Retrospective	29	32	NR	ICA, MCA, AComA, PComA, ACA	NR	NR	NR	0.3 mg/kg
Kırıs, 2024 [19]	Turkey	Prospective	38	48	10/38	ICA, MCA, AComA, PComA, ACA	5.3 ± 2.1	52.6 ± 11.03	13/25	25 mg
Rennert, 2019 [32]	United States	Retrospective	10	10	7/3	Anterior, posterior circulation	NR	55.9 ± 14.8	2/8	0.2 mg/kg
Nakagawa, 2017 [25]	Japan	Retrospective	14	30	NR	ICA, MCA	NR	61.58 ± NR	17/20	25 mg dissolved in 5 ml of 0.9% saline
Munakomi, 2018 [23]	Nepal	Retrospective	40	40	40/0	AComA	NR	NR	NR	NR

*ICG* Indocyanine Green, *NR* Not Reported, *ICA* Internal Carotid Artery, *MCA* Middle Cerebral Artery, *AComA* Anterior Communicating Artery, *PComA* Posterior Communicating Artery, *BA* Basilar Artery, *PCA* Posterior Cerebral Artery, *AChoA* Anterior Choroidal Artery, *AICA* Anterior Inferior Cerebellar Artery, *PICA* Posterior Inferior Cerebellar Artery, *SCA* Superior Cerebellar Artery, *ACA* Anterior Cerebral Artery, *ACP* Anterior Choroidal Plexus, *Sex (M/F)* Male/Female

Also, verification practices were heterogeneous across studies, and only a minority performed independent confirmation (Supplementary Table 2). Ye et al. [43] and Goertz et al. [10] conducted partial postoperative DSA/CTA verification of aneurysm clipping findings, whereas the remaining studies relied exclusively on intraoperative ICG-VA + FLOW 800. Because verification was incomplete and inconsistent, false negatives cannot be excluded, and the true misclipping rate is likely underestimated. Furthermore, several included studies descriptively reported potential utility of the combined use of ICG-VA + FLOW 800 in anterior circulation aneurysms, particularly AComA, MCA, and ICA, where anatomical complexity limits the visual interpretation of ICG-VA [3, 10, 19]. In these cases, FLOW 800 was used for intraoperative perfusion assessment, identification of clip-induced stenosis, and evaluation of critical perforators, supporting intraoperative assessment and surgical decision-making [10, 41]. Some studies also reported its application in posterior circulation aneurysms, allowing confirmation of sac occlusion and patency of the main vessel even in deep fields [10, 18, 41]. ICG-VA showed significant limitations such as dye persistence, attenuation due to calcification, and poor visualization of perforators [20, 27, 36], several included studies reported that FLOW 800 helped differentiate real flow from residual fluorescence and assisted in the identification of incomplete clipping [19, 36]. Although the dynamic parameters of the FLOW 800 combination do not differ between ruptured and unruptured aneurysms, some studies reported potential clinical utility in ruptured aneurysms due to the compromised visual field and the markedly higher risk of postoperative infarction [21, 24]. Finally, as descriptively reported in individual studies, the combined use of ICG-VA and FLOW 800 was considered useful in giant, calcified, or previously treated aneurysms for intraoperative assessment of occlusion, vessel patency, subtle stenoses, and vascular reconstructions or bypasses [10, 19, 32, 41]. 

However, due to limited subgroup-specific reporting and the absence of formal comparative subgroup analyses, these observations should be interpreted cautiously.

This table summarizes the main methodological and clinical characteristics of the included studies, including study design, number of aneurysms, rupture status, anatomical location, aneurysm size, patient demographics, and ICG dosage.

### Misclipping

**Fig. 2:**
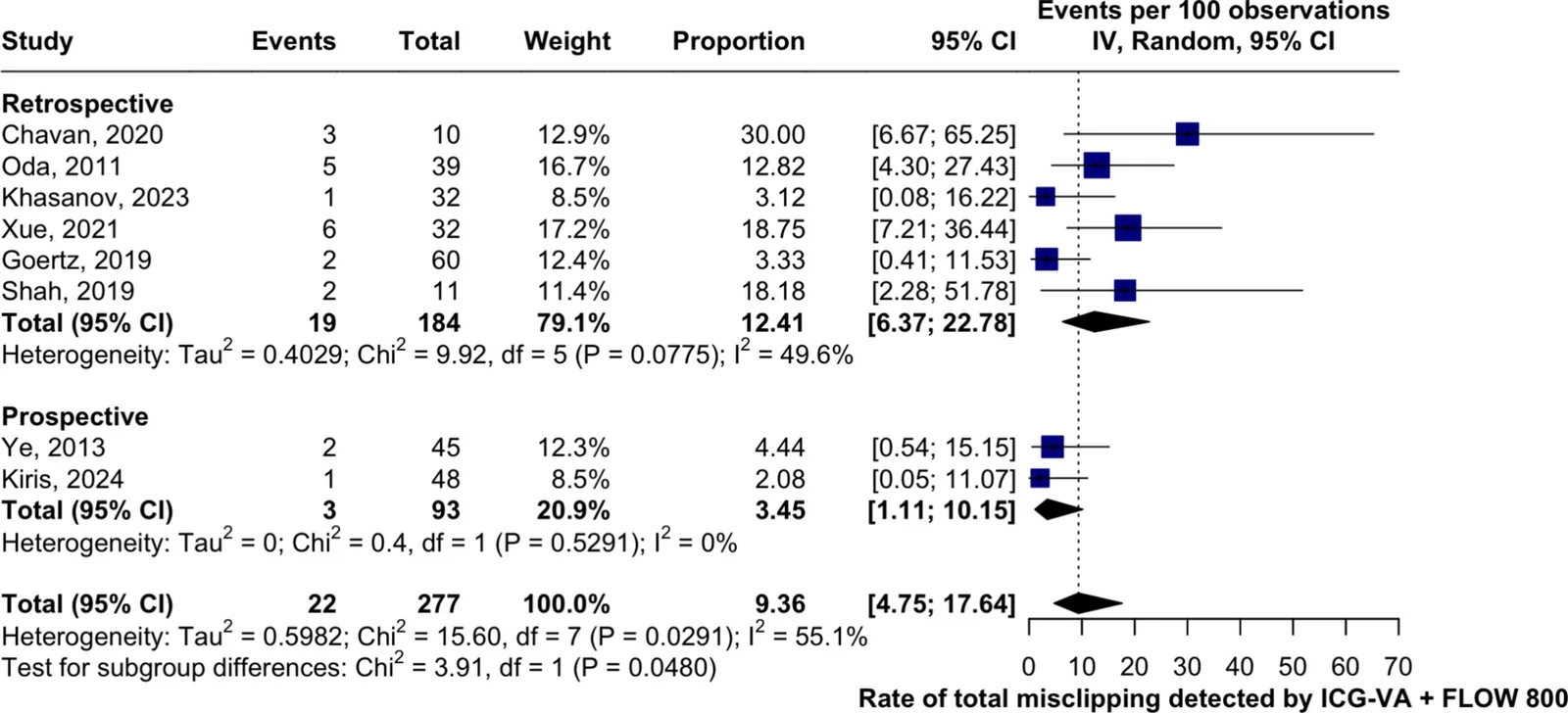
Forest plot of the rate of misclippings identified by ICG-VA + FLOW 800 using a random-effects model (*n* = aneurysms). Blue squares represent individual study weights; larger squares indicate greater statistical weight. The diamond indicates the pooled estimate. Pooled proportions are reported with 95% confidence intervals (CI), heterogeneity is expressed with I^2^ and τ.^2^

A total of eight studies (*n* = 277 aneurysms) evaluated intraoperative misclipping detection using combined ICG-VA and FLOW 800 (see Fig. 2). The pooled misclipping rate was 9.36% (95% CI: 4.75–17.64; I^2^ = 55.1%, τ^2^ = 0.60)**.**When stratified by study design, retrospective studies showed a higher pooled rate of 12.41% (95% CI: 6.37–22.78; I^2^ = 49.6%, τ^2^ = 0.40) compared with prospective studies, which had a pooled rate of 3.45% (95% CI: 1.11–10.15; I^2^ = 0%, τ^2^ = 0)**.** Finally, the Leave-One-Out sensitivity analysis showed that no study significantly modified the pooled proportion of misclipping. Furthermore, the funnel plot presented marked asymmetry, which could indicate the presence of publication bias or the influence of atypical studies (see Supplementary Figs. 1 and 2).

### Aneurysm remnant

**Fig. 3:**
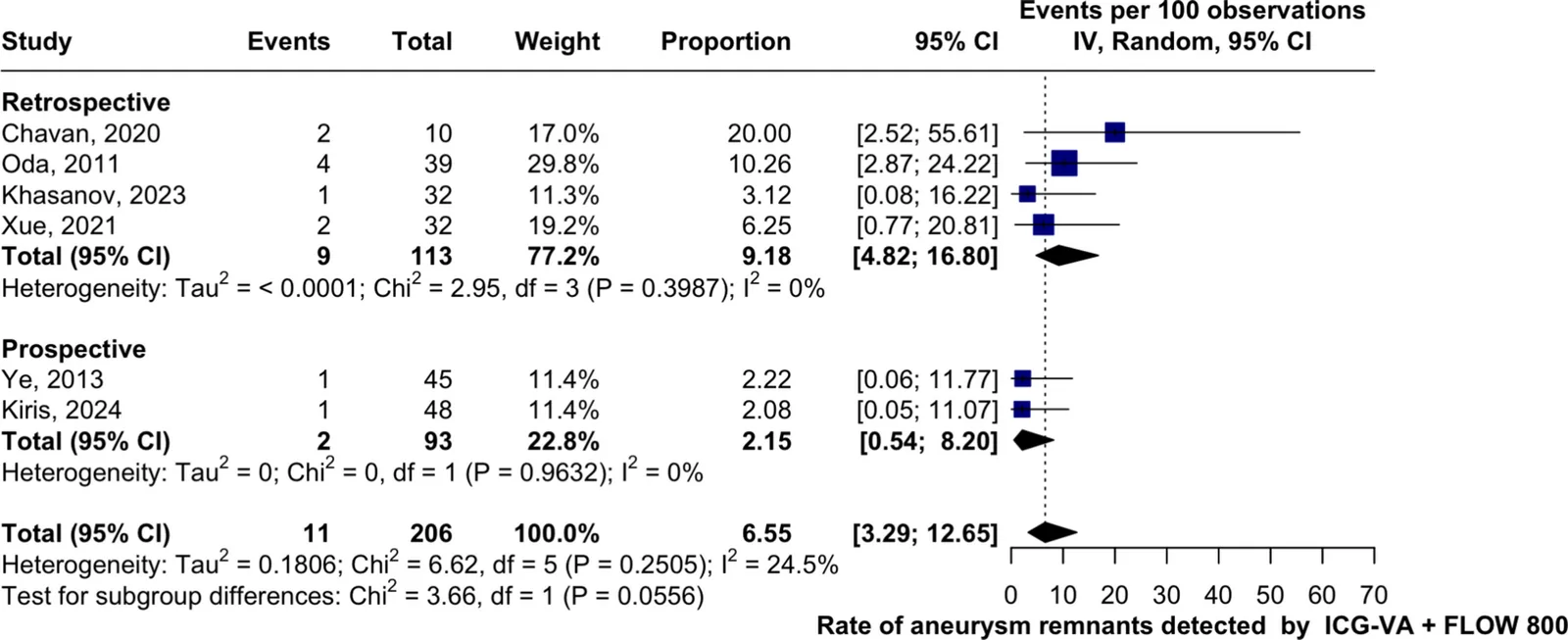
Forest plot of the rate of aneurysm remnants identified by ICG-VA + FLOW 800 using a random-effects model (*n* = aneurysms). Blue squares represent individual study weights; larger squares indicate greater statistical weight. The diamond indicates the pooled estimate. Pooled proportions are reported with 95% CI; heterogeneity is expressed with I^2^ and τ.^2^

A total of six studies (*n* = 206 aneurysms) evaluated intraoperative detection of aneurysmal remnants using combined ICG-VA and FLOW 800 (see Fig. 3). The pooled proportion was 6.55% (95% CI: 3.29–12.65; I^2^ = 24.5%, τ^2^ = 0.18). When stratified by study design, retrospective studies showed a higher detection proportion 9.18% (95% CI: 4.82–16.80; I^2^ = 0%, τ^2^ = 0) compared with prospective studies 2.15% (95% CI: 0.54–8.20; I^2^ = 0%, τ^2^ = 0). Additionally, leave-one-out sensitivity analysis showed no significant changes when excluding each study individually (see Supplementary Image 3). The funnel plot showed slight asymmetry, which could suggest the existence of publication bias (see Supplementary Image 4).

### Vessel stenosis/occlusion

**Fig. 4:**
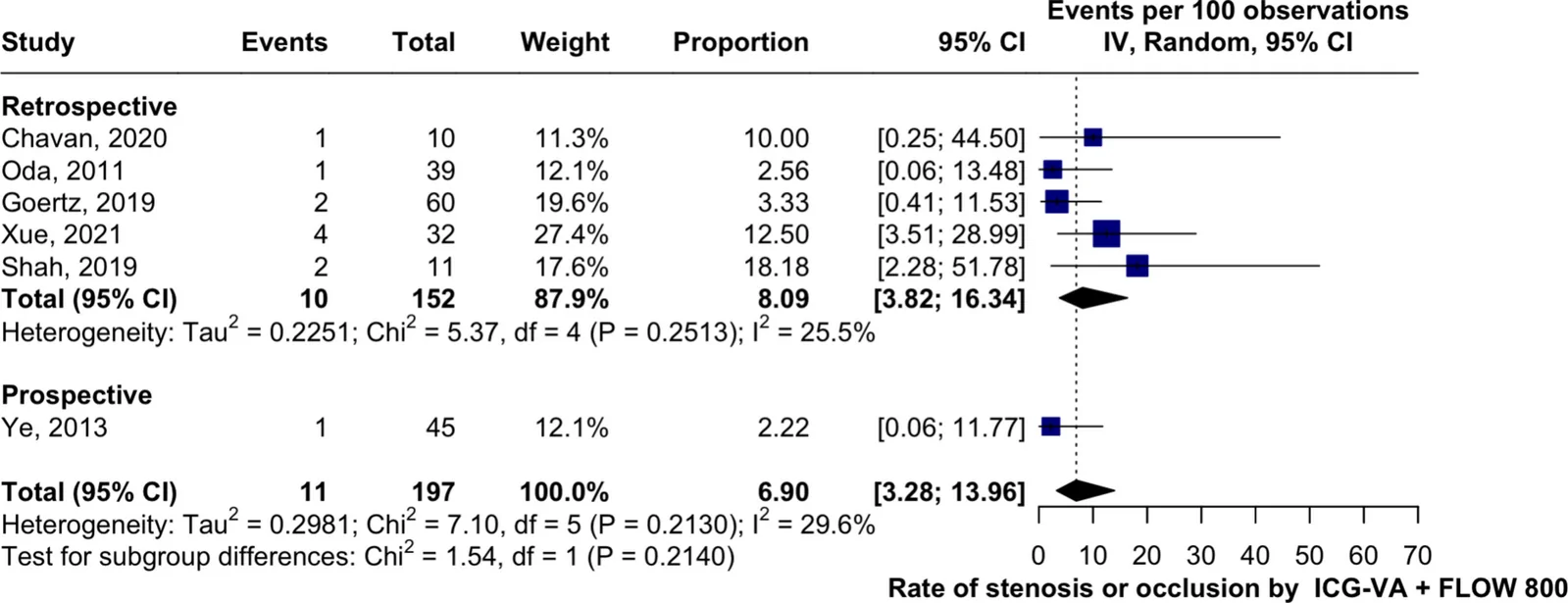
Forest plot of the rate of vessel stenosis/occlusion identified by ICG-VA + FLOW 800 using a random-effects model (*n* = aneurysms). Blue squares represent individual study weights; larger squares indicate greater statistical weight. The diamond indicates the pooled estimate. Pooled proportions are reported with 95% CI; heterogeneity is expressed with I^2^ and τ.^2^

A total of six studies (*n* = 197 aneurysms) evaluated intraoperative detection of vascular stenosis or occlusion by the combined use of ICG-VA and FLOW 800 (see Fig. 4). The pooled proportion was 6.90% (95% CI: 3.28–13.96; I^2^ = 29.6%, τ^2^ = 0.30). When stratified by study design, retrospective studies showed a proportion of 8.09% (95% CI: 3.82–16.34; I^2^ = 25.5%, τ^2^ = 0.23), while the only included prospective study reported a proportion of 2.22% (95% CI: 0.06–11.77; I^2^ = 0%, τ^2^ = 0.30). Leave-one-out sensitivity analysis showed that individual exclusion of studies did not substantially change the pooled proportion (see Supplementary Image 5). Furthermore, the funnel plot revealed a slight asymmetry, which could indicate the presence of publication bias (see Supplementary Image 6).

### Clip repositioning

**Fig. 5:**
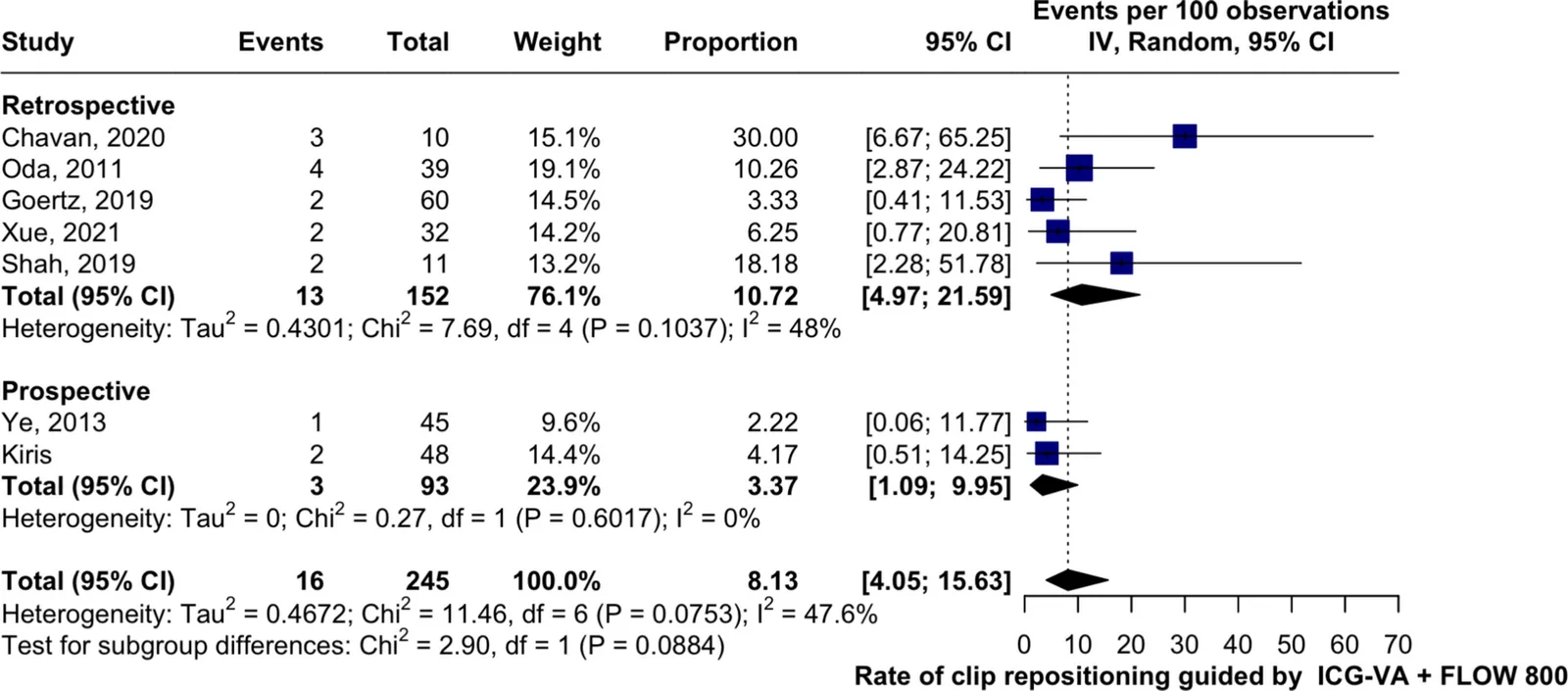
Forest plot of the rate of clip repositioning identified by ICG-VA + FLOW 800 using a random-effects model (*n *= aneurysms). Blue squares represent individual study weights; larger squares indicate greater statistical weight. The diamond indicates the pooled estimate. Pooled proportions are reported with 95% CI; heterogeneity is expressed with I^2^ and τ.^2^

A total of seven studies (*n* = 245 aneurysms) evaluated intraoperative clip repositioning prompted by ICG-VA and FLOW 800 findings (see Fig. 5). The pooled event rate was 8.13% (95% CI: 4.05–15.63; I^2^ = 47.6%, τ^2^ = 0.47). When stratified by study design, retrospective studies showed a higher repositioning rate 10.72% (95% CI: 4.97–21.59; I^2^ = 48%, τ^2^ = 0.43), compared with prospective studies, which reported a rate of 3.37% (95% CI: 1.09–9.95; I^2^ = 0%, τ^2^ = 0.27**).** Leave-one-out sensitivity analysis revealed that individual study omission did not significantly alter the pooled rate (see Supplementary Image 7). Furthermore, the funnel plot showed a slight asymmetry, suggesting the possible presence of publication bias (see Supplementary Image 8).

A summary table is presented showing the combined proportions of intraoperative events detected using ICG-VA and FLOW 800, including the number of studies, sample size, confidence intervals, and I^2^ for each outcome evaluated (Table 2).

**Table 2:** Summary of combined proportions for intraoperative events detected using ICG-VA + FLOW 800

Intraoperative Event Detected	Number of Studies	Total Aneurysms	Pooled Proportion (%)	95% CI	I^2^ (%)
Misclipping	8	277	9.36	4.75–17.64	55.1
Aneurysm remnant	6	206	6.55	3.29–12.65	24.5
Vascular stenosis or occlusion	6	197	6.90	3.28–13.96	29.6
Clip repositioning	7	245	8.13	4.05–15.63	47.6

All totals and pooled proportions are reported per aneurysm (*n* = aneurysms)

### Adverse effects

Most studies reported no significant adverse effects from the intraoperative use of indocyanine green (ICG). Several authors explicitly stated the absence of adverse reactions. Although no clinical complications were described, some studies mentioned technical limitations, such as visualization difficulties in thick-walled aneurysms or anatomical interferences.

### Quality assessment

**Fig. 6:**
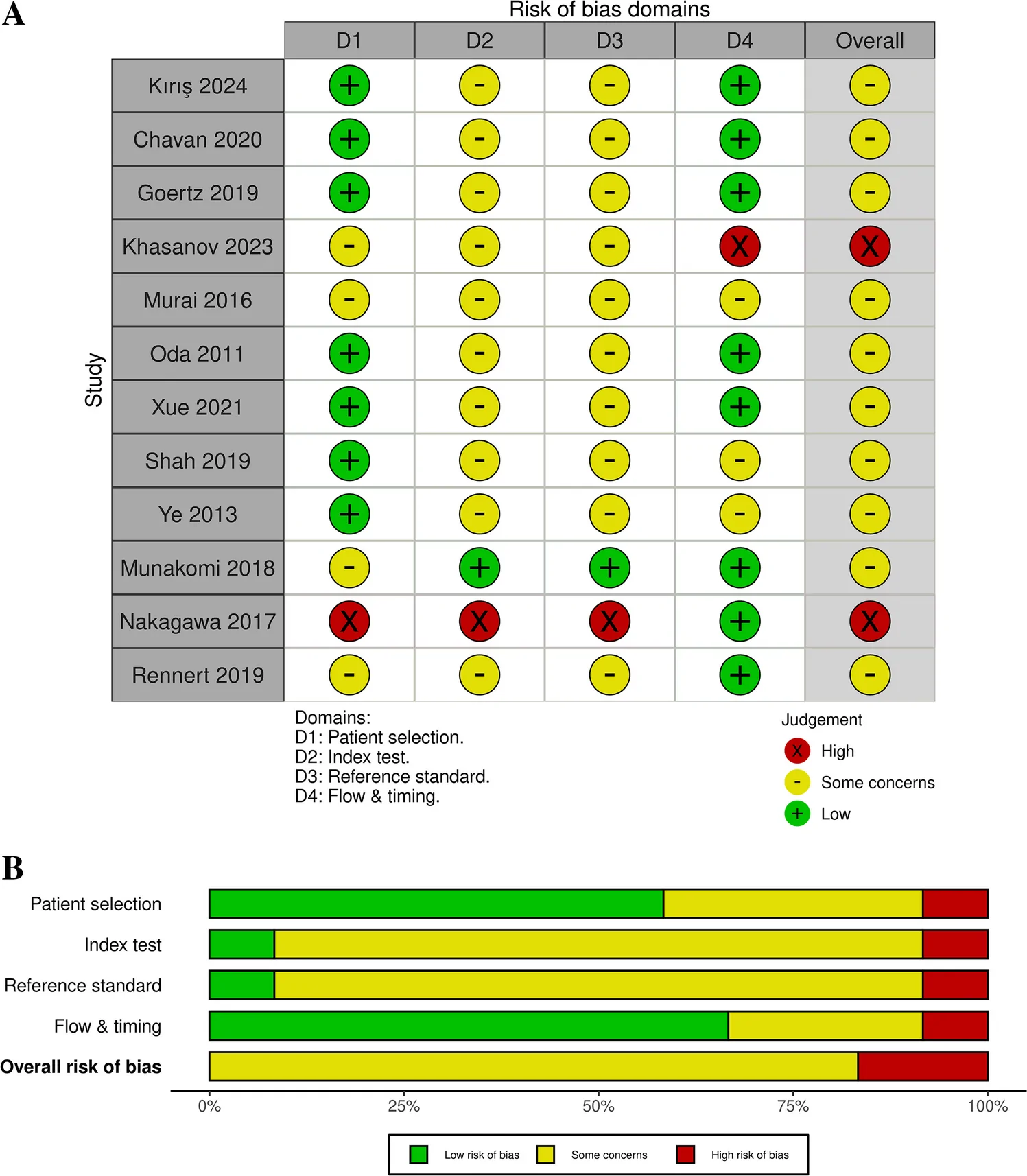
Risk of bias assessment using the QUADAS-2 tool. Panel A shows the risk of bias per study across four domains: patient selection, index test, reference standard, and flow & timing. Panel B summarizes the percentage of judgments across all studies. Green indicates low risk; yellow indicates some concerns and red indicates high risk of bias

Although no study was free of concerns in all domains, most studies (66.7%) were classified as having only some methodological concerns rather than high risk. The most frequent sources of bias were related to the index test and reference standard, which often lacked sufficient methodological detail. Only two studies demonstrated a high risk of bias, particularly Nakagawa 2017 with high risk in three domains. These results suggest a moderate overall quality, highlighting the need for better reporting and standardization in future diagnostic accuracy studies involving ICG-VA and FLOW 800 (Fig. 6).

### Meta-regression

**Fig. 7:**
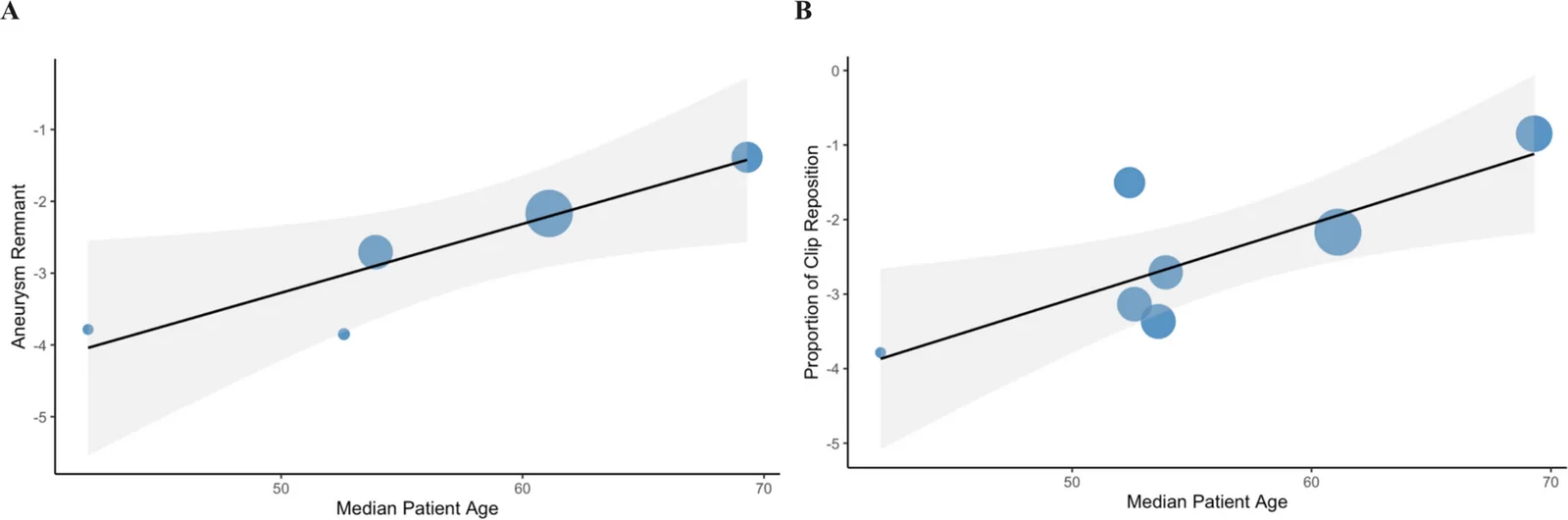
Meta-regression bubble plots showing the association between median patient age and intraoperative outcomes.** A** Aneurysm remnant was significantly associated with age (*p* = 0.0247). **B** Clip repositioning was significantly predicted by age (*p* = 0.0073). Bubble size reflects study weight in the meta-regression model. Shaded areas represent 95% confidence intervals

Meta-regression did not identify significant predictors for misclipping, aneurysm remnant, or vascular stenosis/occlusion when year of publication was used as a covariate (*p* > 0.05 in all cases). In contrast, mean patient age was a significant predictor for the outcomes of aneurysm remnant (*p* = 0.0247) and clip repositioning (*p* = 0.0073). Furthermore, study design showed a statistically significant association with all clinical outcomes assessed (*p* < 0.0001) (see Fig. 7). See Table 3 for detailed coefficients and for additional meta-regression analyses (see Supplementary Figure S9-S18).

**Table 3:** Predictors of outcome identified on meta-regression

Outcome	Predictor	No. of studies reporting outcome	Total no. of aneurysm analyzed	P value	Significant	R^2^ (%)	I^2^ (%)	τ^2^
Misclipping	Publication year	8	277	0.7486	No	0.0	62.72	0.8291
	Median patient age	7	245	0.0679	No	48.37	42.11	0.2985
	Study design	8	277	< 0.0001	Yes	0.0	40.84	0.3016
Aneurysm remnant	Publication year	6	206	0.5755	No	0.0	32.62	0.3538
	Median patient age	5	174	0.0247	Yes	100	0.0	0.0
	Study design	6	206	< 0.0001	Yes	0.0	0.0	0.0
Vascular stenosis/occlusion	Publication year	6	197	0.2835	No	1.30	84.43	3.9216
	Median patient age	6	197	0.6625	No	0.00	42.49	0.4343
	Study design	6	197	< 0.0001	Yes	0.0	27.26	0.2251
Clip repositioning	Publication year	7	245	0.9562	No	0.0	56.18	0.7113
	Median patient age	7	245	0.0073	Yes	100	0.0	0.0
	Study design	7	245	< 0.0001	Yes	0.0	37.85	0.3050

### Certainty of evidence

According to the GRADE approach, the certainty of evidence for intraoperative outcomes assessed with ICG-VA and FLOW 800 ranged from moderate to low (Table 4). Misclipping and clip repositioning were rated as moderate certainty, supported by acceptable precision, non-serious risk of bias, and moderate heterogeneity (I^2^ = 55.1% and 47.6%). In contrast, aneurysm remnant and vascular stenosis/occlusion were rated as low certainty, downgraded due to serious imprecision reflected in wide confidence intervals, despite showing low heterogeneity (I^2^ = 24.5% and 29.6%). Publication bias was suspected for all endpoints; however, interpretation is limited given the small number of studies (< 10 per outcome).

**Table 4:** Certainty of evidence according to the GRADE framework

Outcome	Risk of Bias	Inconsistency (I^2^)	Indirectness	Imprecision	Publication Bias	Overall Certainty
Misclipping	Not serious	Serious (55.1%)	Not serious	Not serious	Suspected	⬤⬤⬤◯ Moderate
Aneurysm remnant	Not serious	Not serious (24.5%)	Not serious	Serious	Suspected	⬤⬤◯◯ Low
Vascular stenosis/occlusion	Not serious	Not serious (29.6%)	Not serious	Serious	Suspected	⬤⬤◯◯ Low
Clip repositioning	Not serious	Serious (47.6%)	Not serious	Not serious	Suspected	⬤⬤⬤◯ Moderate

This table summarizes the certainty of evidence for each intraoperative outcome assessed using ICG-VA and FLOW 800, based on GRADE criteria: risk of bias, inconsistency, indirectness, imprecision, and potential publication bias. Certainty levels are represented as symbols (⬤⬤⬤⬤ = high; ⬤⬤⬤◯ = moderate; ⬤⬤◯◯ = low; ⬤◯◯◯ = very low)

## Discussion

Our meta-analysis revealed that the combined use of ICG-VA and FLOW 800 allowed the detection of intraoperative misclipping in 9.36% of treated aneurysms. When the mechanisms were disaggregated, aneurysmal remnants were identified in 6.55% of cases (I^2^ = 24.5%) and vascular stenosis or occlusion in 6.90% (I^2^ = 29.6%), suggesting that these errors are due to different mechanisms during clipping. Furthermore, it was observed that in 8.13% of aneurysms, surgical clipping was required after hemodynamic alterations were detected with the combined use of ICG-VA and FLOW 800. Misclipping is a critical cause of surgical failure, either due to remnants or stenosis, with a risk of ischemia or recurrence [35]. Thus, Riva et al. [33] reported 6.1% of misclipping detected with ICG-VA and an additional 4.5% only identified by DSA, our results showed 9.36%, which supports the use of complementary intraoperative assessment methods. In the contemporary surgical landscape, microsurgical clipping is increasingly reserved for aneurysms with complex morphology such as wide-necks, calcification, large size, or recurrence after coiling. This selective case mix underscores the relevance of FLOW 800 for detecting subtle hemodynamic compromise in anatomically challenging lesions.

The presence of aneurysmal remnants after microsurgical clipping is usually associated with wide-necks, complex morphology, or bifurcation location, conditions in which surgical visualization is limited [5]. For example, in the study by Dellaretti et al. [5], 13.3% of aneurysms presented remnants, being more frequent in large aneurysms (40%) and in those that required a greater number of clips, suggesting that complex anatomy increases the risk of incomplete clipping. Concordantly, Won et al. [40] reported that more than 20% of wide-neck aneurysms presented remnants after conventional clipping. In our series, remnants were identified in 6.55% of cases (I^2^ = 0%). This pattern was also observed in studies that included larger average aneurysms, such as that of Xue et al. [41] with 16.8 mm, reinforcing the idea that more complex anatomies can make clipping difficult and increase the risk of technical errors. Intraoperative stenosis detection represents another technical challenge. In the study by Xue et al. [41], 12.5% of aneurysms presented stenosis detected and corrected exclusively by FLOW 800, with no clear abnormalities reported on standard ICG-VA assessment. Similarly, Chavan et al. [3] reported a stenosis correction in 10% of cases, also guided solely by FLOW 800. In our series, the rate was 6.90% (I^2^ = 29.6%). Furthermore, the dose of indocyanine green (ICG) used varied considerably among the included studies, which could influence the diagnostic sensitivity against hemodynamic alterations. This variability, coupled with the limited penetration of ICG (~ 2 mm), restricts the visualization of deep residual flow or partially hidden branches, reported by Raabe et al. [29].

In addition to ICG-VA and FLOW 800, other intraoperative tools have been developed to complement vascular assessment. Perivascular ultrasound flowmetry allows an absolute quantitative measurement of flow in the parent arteries, unlike FLOW 800, which only offers semi quantitative information; it is particularly useful in distal aneurysms or when there is suspicion of hemodynamic insufficiency not evident with ICG-VA [2]. Intraoperative DSA remains the gold standard for confirming complete aneurysm exclusion and vessel patency, surpassing FLOW 800 in the detection of remnants or subtle stenoses; however, its use is restricted to centers with a hybrid operating room and specialized personnel, so it is reserved for complex cases [4, 6]. Finally, neurophysiological monitoring is routinely used to detect intraoperative ischemia and provide functional feedback, although it only offers indirect and complementary information that FLOW 800 does not provide [34]. The utility of FLOW 800 is not limited to detection, but has also demonstrated a direct impact on surgical correction. In the study by Murai et al. [24], areas of hypoperfusion not visible with ICG-VA were identified, leading to clip repositioning or intraoperative adjustments in all cases. Shah et al. [36] documented clip repositioning in 18.1% of aneurysms after detecting flow abnormalities exclusively with FLOW 800. Similarly, Goertz et al. [10] reported intraoperative hypoperfusion findings that prompted surgical adjustments during aneurysm clipping. In our series, the clip was repositioned in 8.13% of cases after hemodynamic findings were detected with ICG-VA + FLOW 800.

Furthermore, the use of FLOW 800 as a complementary tool has been clinically useful in various neurosurgical settings. In cerebral revascularization, Guo et al. [12] reported that it predicted hypoperfusion in 32% of patients with Moyamoya disease, while Du et al. [7] found that prolonged perfusion time measured with FLOW 800 was associated with a three-fold increased risk of hyperperfusion syndrome, reinforcing its value as an intraoperative predictive tool. Additionally, in arteriovenous malformation surgery, Grzyb and Church [11] documented that the combined use of ICG-VA + FLOW 800 allowed the detection of deep vascular nests in 57% of cases. Likewise, Yang et al. [42] demonstrated that, when this technology was integrated with neuronavigation, FLOW 800 contributed to reducing intraoperative bleeding and improving functional recovery in a series of 90 patients. Finally, in tumor surgery, Acerbi et al. [1] demonstrated its usefulness in 93 procedures, where FLOW 800 guided vascular dissection and allowed early detection of perfusion deficits, increasing the overall safety of the approach. Together, these findings suggest that FLOW 800 may provide complementary intraoperative hemodynamic information during aneurysm surgery.

In our study, meta-regression showed that study design was a significant predictor for all outcomes (*p* < 0.0001) and that mean patient age was associated with a higher frequency of aneurysmal remnants (*p* = 0.0247) and need for clip repositioning (*p* = 0.0073). In contrast, year of publication did not show a significant association. For example, Świątnicki et al. [37] found that older age increased the risk of intraoperative rupture (27%), and that large or irregularly domed aneurysms were associated with remnants (17.3%) and cerebral ischemia (13.6%), supporting our findings. Additionally, no major complications associated with the use of VA-ICG or FLOW 800 were reported in the included studies. According to the GRADE assessment, the certainty of the evidence was moderate for the outcomes of misclipping and clip repositioning, but low for aneurysm remnants and stenosis/occlusions, due to imprecision and inconsistency of the results. From a global perspective, ICG-VA represents a cost-effective tool, especially in low- and middle-income countries. For example, Ghosh et al. [9] noted that this technique constitutes an affordable alternative to conventional angiography, although its implementation still faces technical and economic barriers. Although both fluorescein and indocyanine can be used with the FLOW 800, the combination with ICG allows for improved assessment of cerebral blood flow. Therefore, it is preferred in aneurysm surgery. Narducci et al. [26] highlighted that, although fluorescein visualizes anatomy well, it does not offer the hemodynamic analysis that ICG does with the FLOW 800.

## Limitations

The main limitations of this meta-analysis include the small number of studies, its predominantly retrospective design, and the heterogeneous definitions of "stenosis," "remnant," and "clip repositioning." Most results were based solely on intraoperative visualization, without systematic confirmation by DSA, CTA, or puncture. Only a few studies performed partial postoperative verification, introducing partial verification bias that likely underestimates the true rates of misclipping by omitting false negatives, while occasionally generating false positives when intraoperative abnormalities normalize on angiography. Inconsistent information on temporary clipping and the timing of ICG-VA/FLOW 800 acquisition may have influenced the detection of perfusion abnormalities. Additionally, limited clinical reporting prevented subgroup analyses by anterior versus posterior circulation, rupture status, or aneurysm morphology, restricting interpretation of potential anatomical or hemodynamic modifiers. Variability in ICG dosing and reinjection protocols across studies may also influence fluorescence intensity and the stability of FLOW 800 curves; standardized dosing (e.g., 0.2–0.3 mg/kg pre- and post-clipping) would improve reproducibility. Although QUADAS-2 was applied, incomplete verification limited risk-of-bias assessment, and GRADE certainty was reduced due to heterogeneity and imprecision.

Because this study was based on a single-arm proportional meta-analysis without direct comparative groups, causal inferences regarding superiority of the combined ICG-VA and FLOW 800 approach over isolated ICG-VA should be interpreted cautiously.

## Conclusion

The combined use of ICG-VA and FLOW 800 may serve as a complementary intraoperative tool for the assessment of misclipping, aneurysm remnants, and vascular compromise during aneurysm surgery. However, it should not be considered a replacement for digital subtraction angiography (DSA), as no direct comparative validation was available. These findings summarize the pooled intraoperative detection rates reported with the combined use of ICG-VA and FLOW 800 in aneurysm surgery. Future high-quality prospective studies are essential to confirm these descriptive intraoperative findings in larger prospective comparative studies.

## Supplementary Information

Below is the link to the electronic supplementary material.

ESM 1 Supplementary Material 1 (PDF 1.04 MB)

## Data Availability

All data analyzed during this study are included in this published article and its supplementary information files.

## Declarations

### Registration

The protocol of this review was registered in PROSPERO (CRD420251014600).
