# Supplementary material for: Efficacy of intraoperative indocyanine green videoangiography (ICG-VA) and FLOW 800 in the surgical management of intracranial aneurysms: a systematic review and meta-analysis
Source: Acta Neurochir (Wien). 2026 Jan 26;168(1):24. doi: 10.1007/s00701-026-06779-6 (PMC12847191; doi:10.1007/s00701-026-06779-6)
Supplement: Supplementary file 1 — Supplementary Material 1 (PDF 1.04 MB) [file 701_2026_6779_MOESM1_ESM.pdf]

SUPPLEMENTAL MATERIAL

Efficacy of Intraoperative Indocyanine Green Videoangiography (ICG-VA) and FLOW 800 in the Surgical Management of Intracranial Aneurysms: A Systematic Review and Meta-Analysis

Authors:
Albert Gabriel Turpo-Peqqueña[1,4,7]; Santiago Alejandro Santos-Vargas[1]; Harlly Romed Loza-Chipa[1,7], Francisco Martins Lamas[2]; George Alejandro Espinoza-Laura[1]; Claudia Solange Núñez-Basurco[1]; Diego Napoleon Medina-Neira[1], Valeria Alejandra Benites-Bustamante[1], Rayza Ruth Osorio-Pacheco[5]; Josue Rodrigo Turpo-Peqqueña[3], Gladys Huanca-Quispe[1,9]; Cristhian Adolfo Vizcarra Vizcarra[1] ; Badhin Gomez Valdez[7] ; Julian Alejandro Rivillas[6,8], Richard Hernández-Mayori[9]

Affiliations:
[1] Faculty of Human Medicine, Universidad Católica de Santa María, Center for Research and Medical Studies (CIEM),Arequipa,Perú
[2] Faculty of Human Medicine, Universidade Federal de Ciências da Saúde de Porto Alegre (UFCSPA), Porto Alegre, Brasil.
[3] Faculty of Human Medicine, Universidad de San Martín de Porres, Arequipa,Perú
[4] Faculty of Biology, National University of San Agustín, Arequipa, Peru.
[5] Faculty of Nursing, Universidad Nacional de San Agustin, Arequipa,Perú
[6] Faculty of Medicine, Université de Montréal, Montreal, Canada
[7] Center for Molecular Engineering Research (CIIM), Universidad Católica de Santa María, Arequipa, Perú
[8] Neurology Service, Valle del Lili Foundation, Cali, Colombia
[9] Neurosurgery Service, Hospital Regional Honorio Delgado Espinoza (HRHDE), Arequipa,Perú

Supplemental Table 1. Exact search strings (updated: 20 February 2025).

Database	Search string
PubMed/MEDLINE	((("Intracranial Aneurysm"[Mesh] OR "intracranial aneurysm"[tiab] OR "cerebral aneurysm"[tiab] OR "brain aneurysm"[tiab] OR "subarachnoid hemorrhage"[tiab] OR SAH[tiab]) OR (aneurysm*[tiab] AND (intracranial[tiab] OR cerebral[tiab] OR brain[tiab]))) AND ("Indocyanine Green"[Mesh] OR "indocyanine green"[tiab] OR ICG[tiab] OR videoangiograph*[tiab] OR "video angiograph*"[tiab] OR "FLOW 800"[tiab] OR fluorescen*[tiab]) AND (clipp*[tiab] OR "microsurgical clipping"[tiab] OR microsurg*[tiab] OR intraoperat*[tiab] OR "intraoperative imaging"[tiab] OR endovascular[tiab])
Results: 381	
CENTRAL (Cochrane Library)	((aneurysm OR aneurysms OR "intracranial aneurysm" OR "cerebral aneurysm" OR "brain aneurysm" OR "subarachnoid hemorrhage" OR SAH) AND ("indocyanine green" OR ICG OR "indocyanine green angiography" OR "fluorescence angiography" OR fluorescen* OR "near infrared" OR "near-infrared" OR angiograph*) AND (intraoperat* OR clipp* OR microsurg*))
Results: 116	
EMBASE	('intracranial aneurysm'/exp OR 'intracranial aneurysm':ti,ab OR 'cerebral aneurysm':ti,ab OR 'brain aneurysm':ti,ab OR aneurysm*:ti,ab)

	AND ('indocyanine green'/exp OR 'indocyanine green':ti,ab OR icg:ti,ab OR videoangiograph*:ti,ab OR fluorescen*:ti,ab OR 'flow 800':ti,ab OR flow800:ti,ab) AND (clipp*:ti,ab OR microsurg*:ti,ab OR intraoperat*:ti,ab OR 'intraoperative imaging':ti,ab OR 'microsurgical clipping':ti,ab)
Results: 136	
SCOPUS	TITLE-ABS-KEY(("intracranial aneurysm" OR "intracranial aneurysms" OR "cerebral aneurysm" OR "cerebral aneurysms" OR "brain aneurysm" OR "brain aneurysms") AND ("indocyanine green" OR ICG OR "FLOW 800" OR FLOW800) AND (clipp* OR "microsurgical clipping" OR microsurg* OR "endovascular treatment"))
Results: 287	
WEB OF SCIENCE	TS=((("intracranial aneurysm" OR "intracranial aneurysms" OR "cerebral aneurysm" OR "cerebral aneurysms" OR "brain aneurysm" OR "brain aneurysms" OR "subarachnoid hemorrhage" OR SAH) AND ("indocyanine green" OR ICG OR "indocyanine green angiography" OR videoangiograph* OR "video angiograph*" OR "FLOW 800" OR FLOW800) AND (clipp* OR "microsurgical clipping" OR microsurg* OR intraoperat* OR "intraoperative imaging" OR endovascular OR "endovascular treatment")))
Results: 292	

Supplementary Table 2. Verification of ICG-VA + FLOW 800 findings across included studies

Study	Verification modality used (DSA / CTA / Puncture)	Independent verification performed	Verification approach	Verification altered interpretation (FP/FN)
Oda, 2011	None	No	None	Not assessable
Ye, 2013	CTA / DSA	Yes	Partial	FN possible
Murai, 2016	None	No	None	Not assessable
Goertz, 2019	CTA / DSA (selected cases)	Yes	Selective	FN not reported
Shah, 2019	None	No	None	Not assessable
Chavan, 2020	None	No	None	Not assessable
Xue, 2021	None	No	None	Not assessable
Khasanov, 2023	None	No	None	Not assessable
Kırış, 2024	None	No	None	Not assessable
Rennert, 2018	CTA/DSA (only for bypass patency; no aneurysm clipping)	Yes	Not applicable (no aneurysm misclipping outcomes)	Not assessable
Nakagawa, 2017	None	No	None	Not assessable
Munakomi, 2018	None	No	None	Not assessable

Abbreviations: DSA: Digital Subtraction Angiography;CTA: Computed Tomography Angiography;FP: False Positive; FN: False Negative.

Supplemental Table 3. Operational Definitions of Outcomes

Outcome	Definition	Example
Aneurysm remnant	Residual aneurysm filling after clipping.	Persistent fluorescence / rising FLOW 800 curve.
Vessel stenosis/occlusion	Reduced or absent flow in a nearby vessel.	Low fluorescence / delayed time-to-peak.
Misclipping	Either remnant or stenosis (counted once if both occur in same aneurysm).	Any abnormal filling or narrowing.
Clip repositioning	Intraoperative adjustment of the clip.	Clip moved after abnormal FLOW 800 pattern.

SUPPLEMENTAL RESULTS

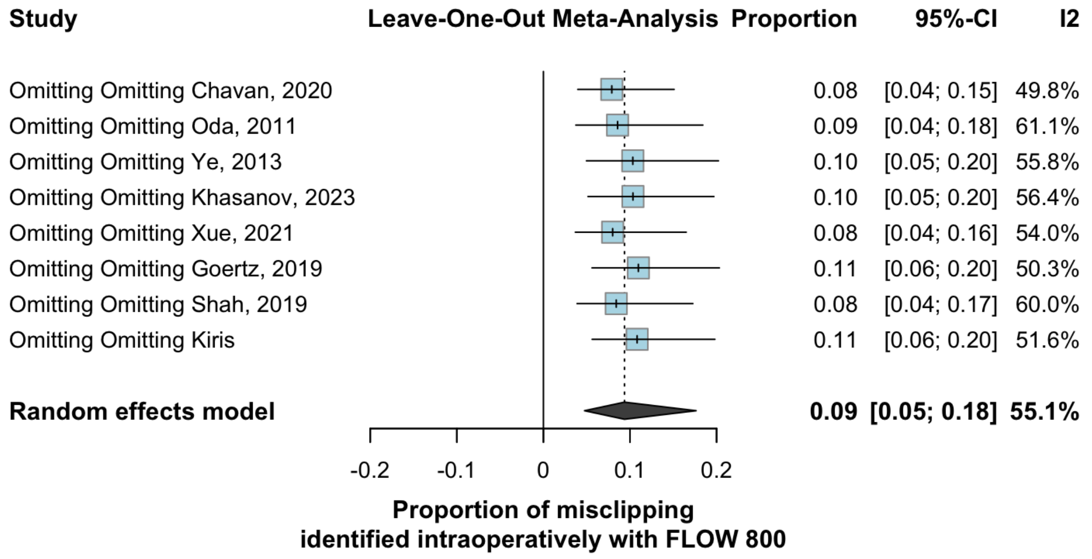

Supplementary Figure 1 – Leave-one-out sensitivity analysis: Proportion of misclipping detected with ICG-VA + FLOW 800

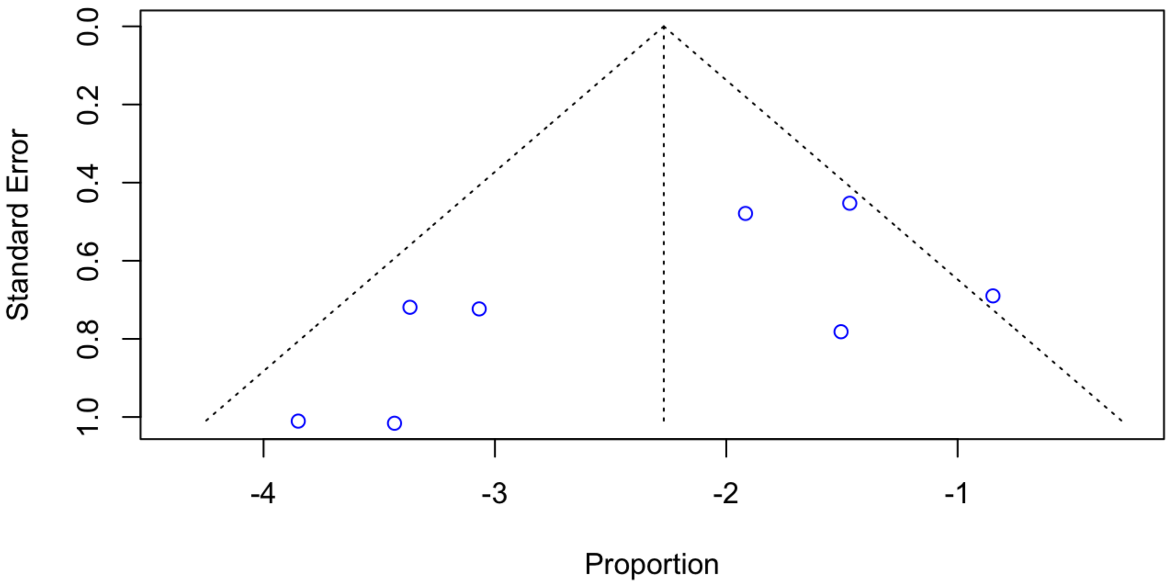

Supplementary Figure 2- Funnel Plot: Proportion of misclipping detected with ICG-VA + FLOW 800

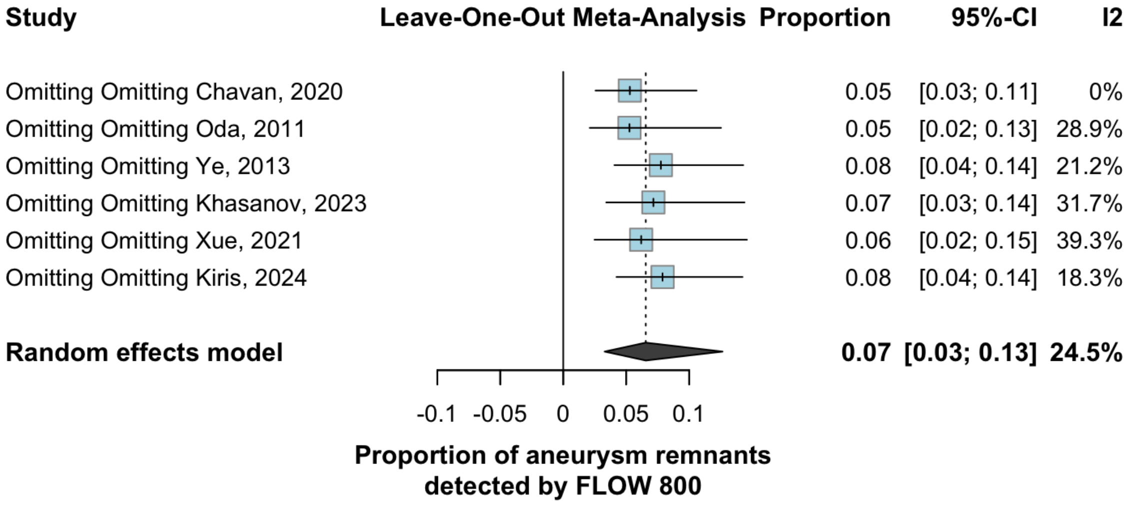

Supplementary Figure 3 – Leave-one-out sensitivity analysis: Proportion of Aneurysm remnants detected with ICG-VA + FLOW 800

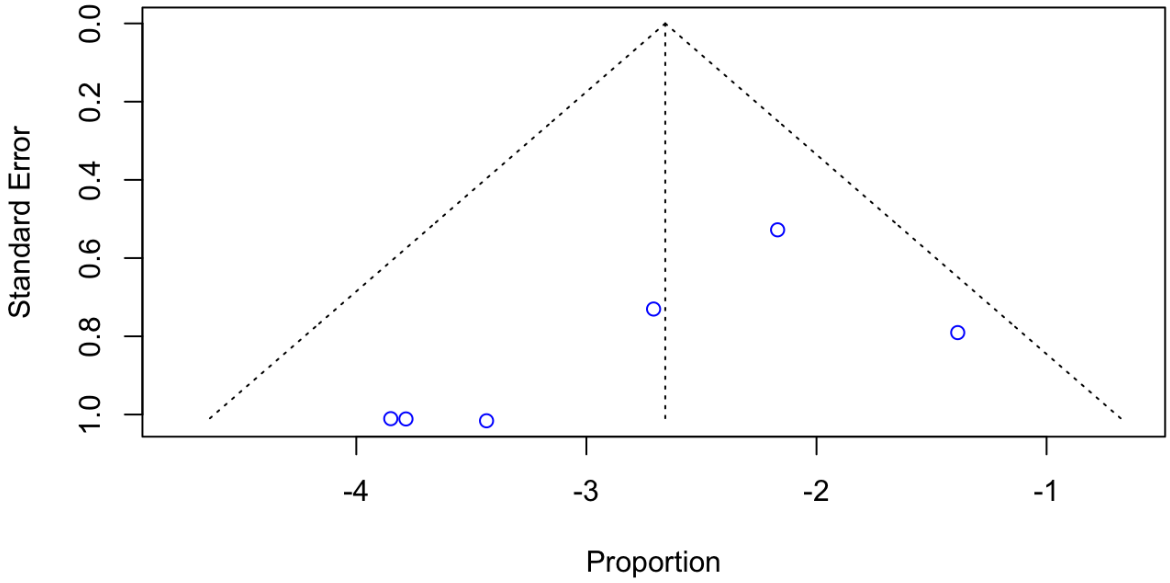

Supplementary Figure 4 - Funnel Plot: Proportion of Aneurysm remnants detected with ICG-VA + FLOW 800

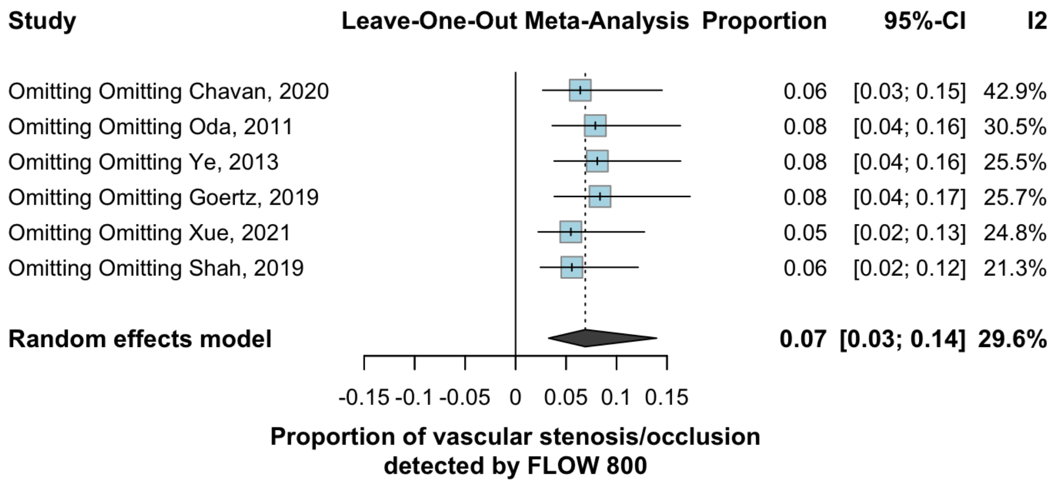

Supplementary Figure 5 – Leave-one-out sensitivity analysis: Proportion of Vascular compromise detected with ICG-VA + FLOW 800

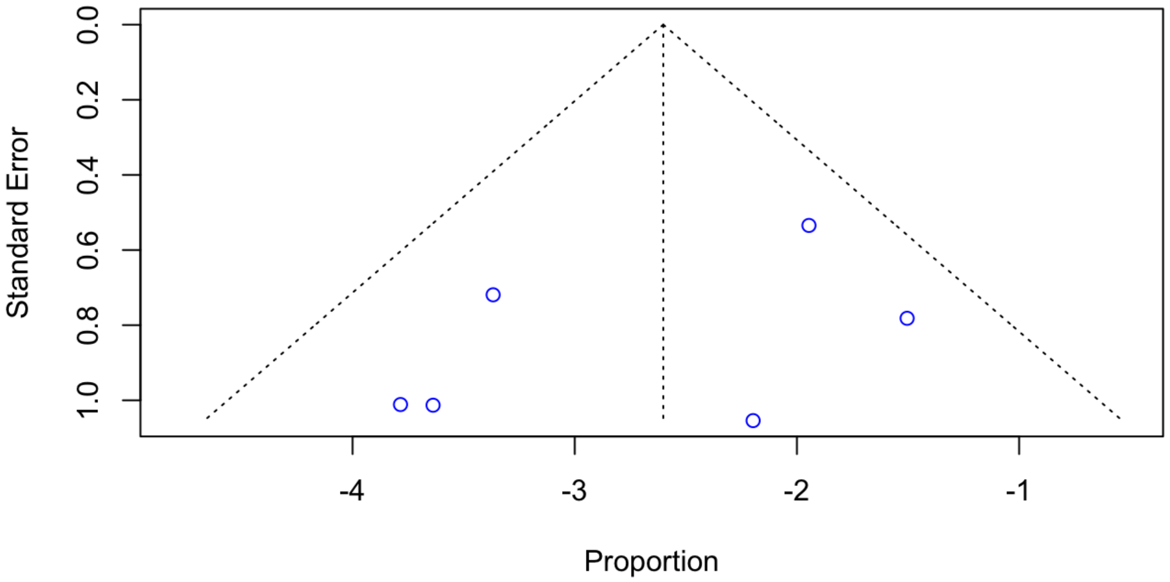

Supplementary Figure 6 - Funnel Plot: Proportion of Vascular compromise detected with ICG-VA + FLOW 800

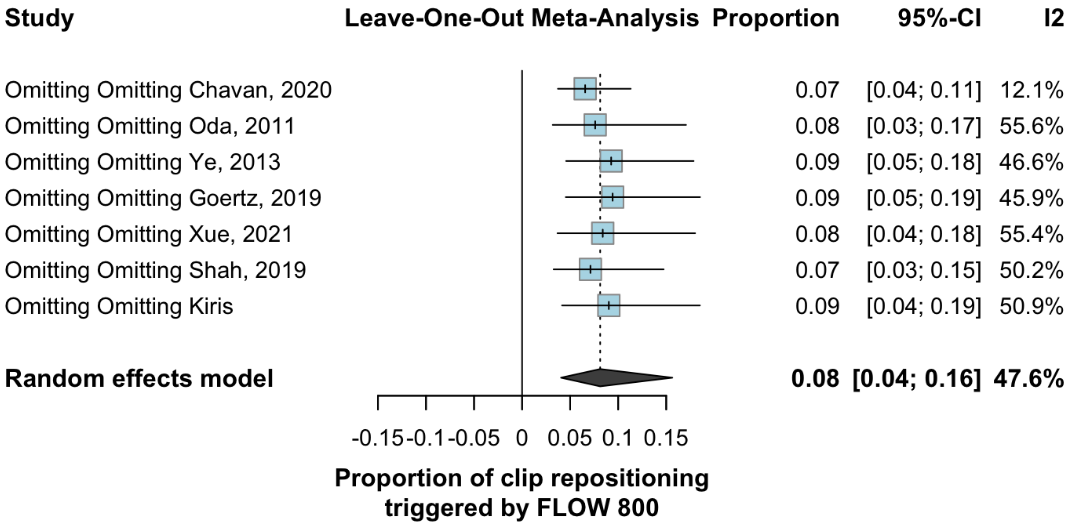

Supplementary Figure 7 – Leave-one-out sensitivity analysis: Proportion of Clip repositioning detected with ICG-VA + FLOW 800

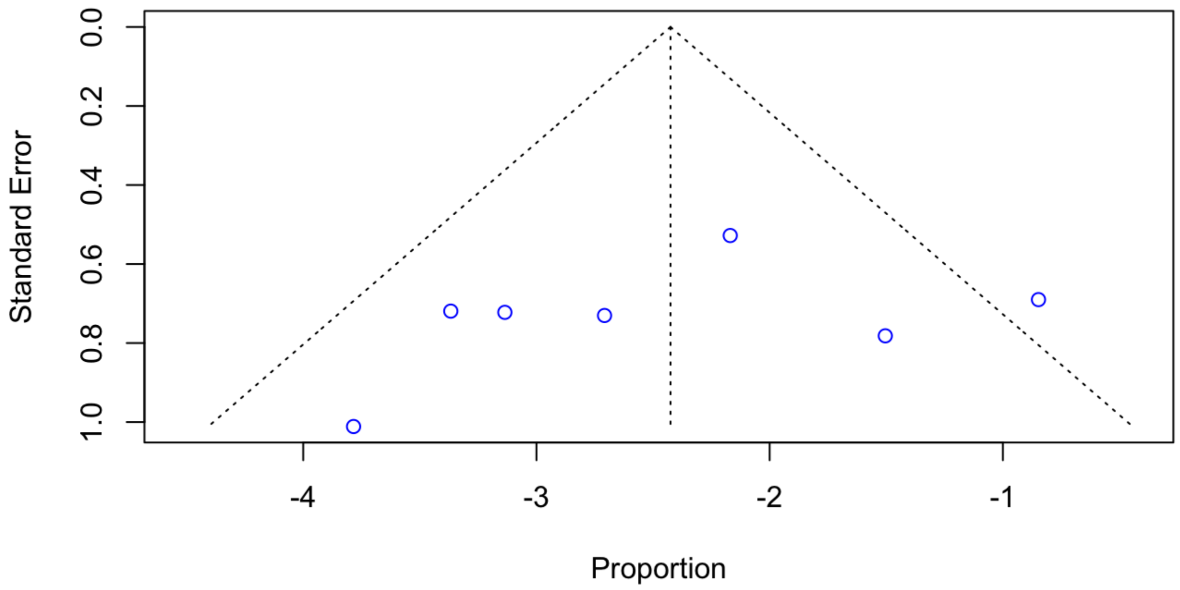

Supplementary Figure 8 - Funnel Plot: Proportion of Clip repositioning detected with ICG-VA + FLOW 800

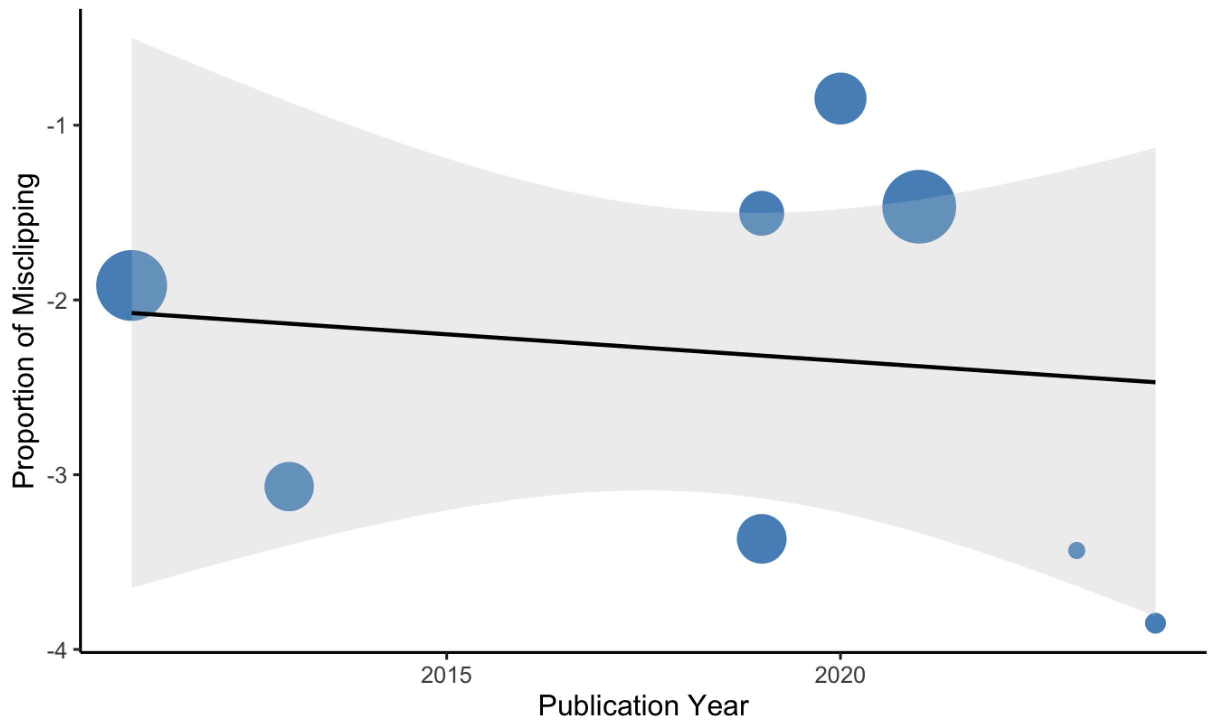

Supplementary Figure 9 – Meta-regression bubble plots: Publication year predicted Misclipping

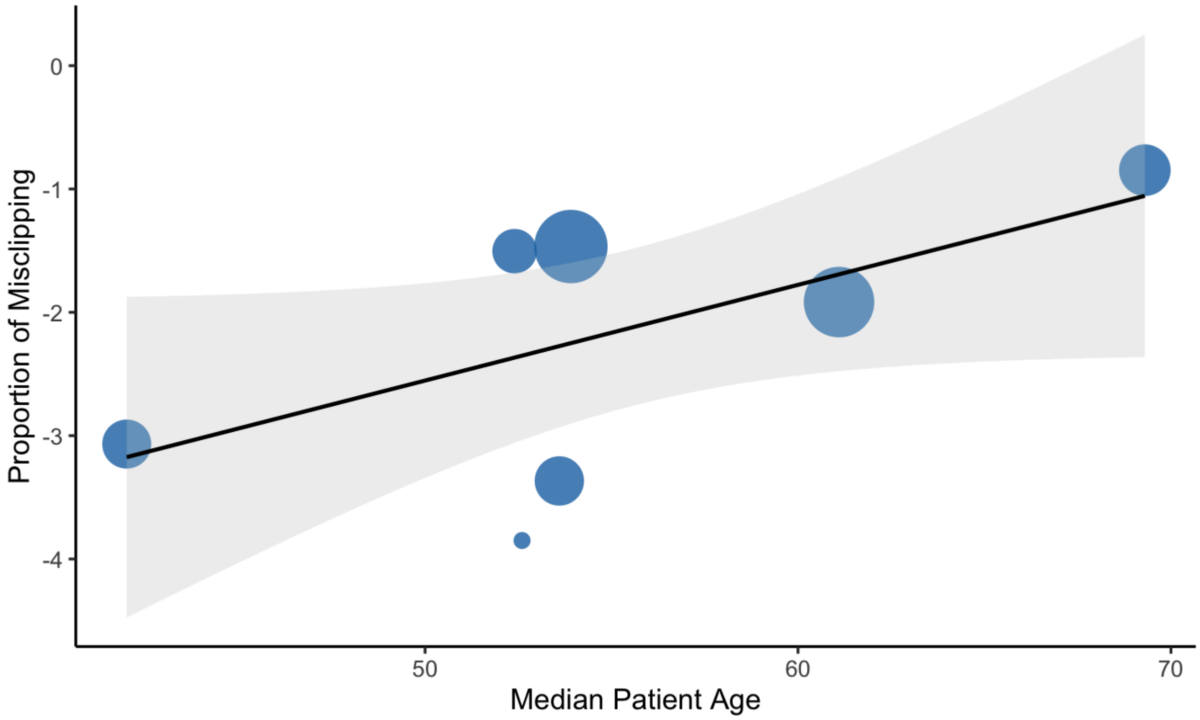

Supplementary Figure 10 – Meta-regression bubble plots: Median Patient Age predicted Misclipping

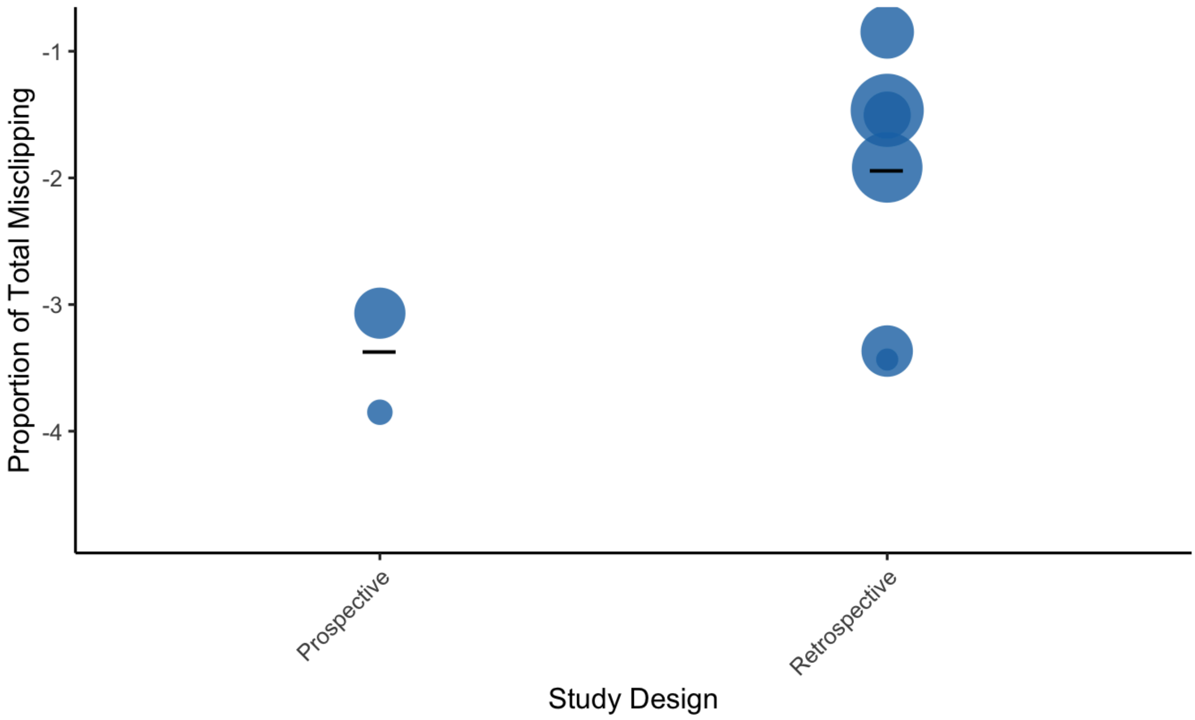

Supplementary Figure 11 – Meta-regression bubble plots: Study Design predicted Misclipping

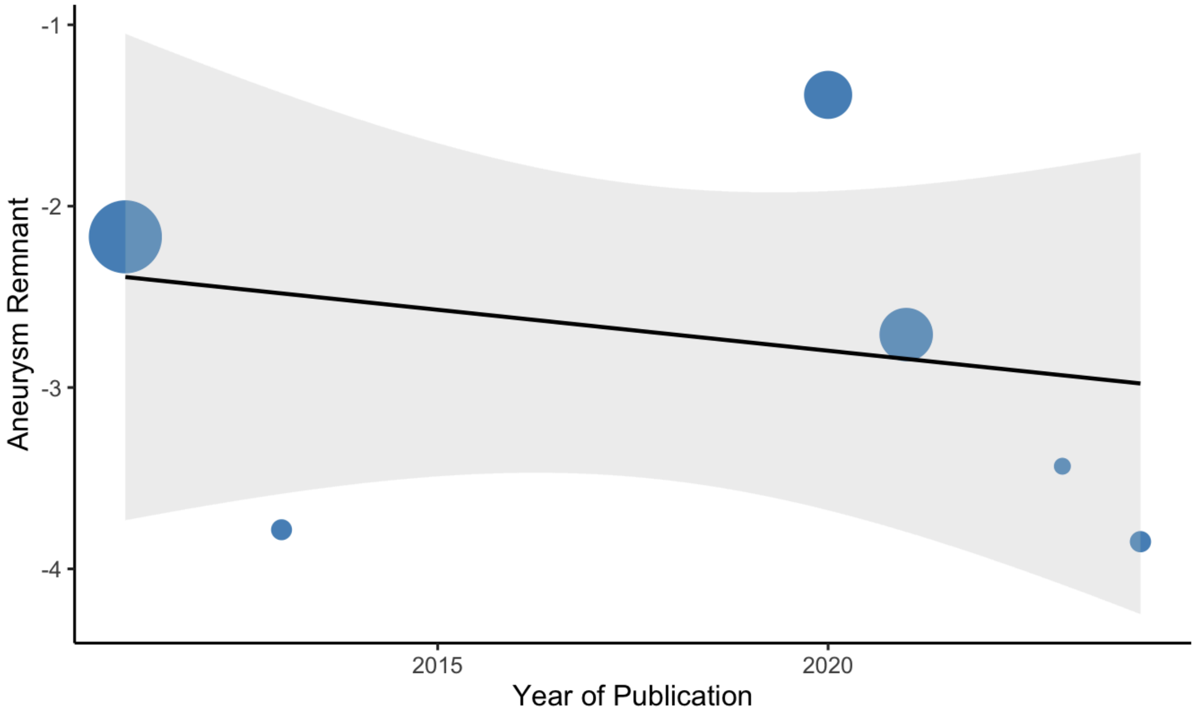

Supplementary Figure 12 – Meta-regression bubble plots: Publication year predicted Aneursym Remnant

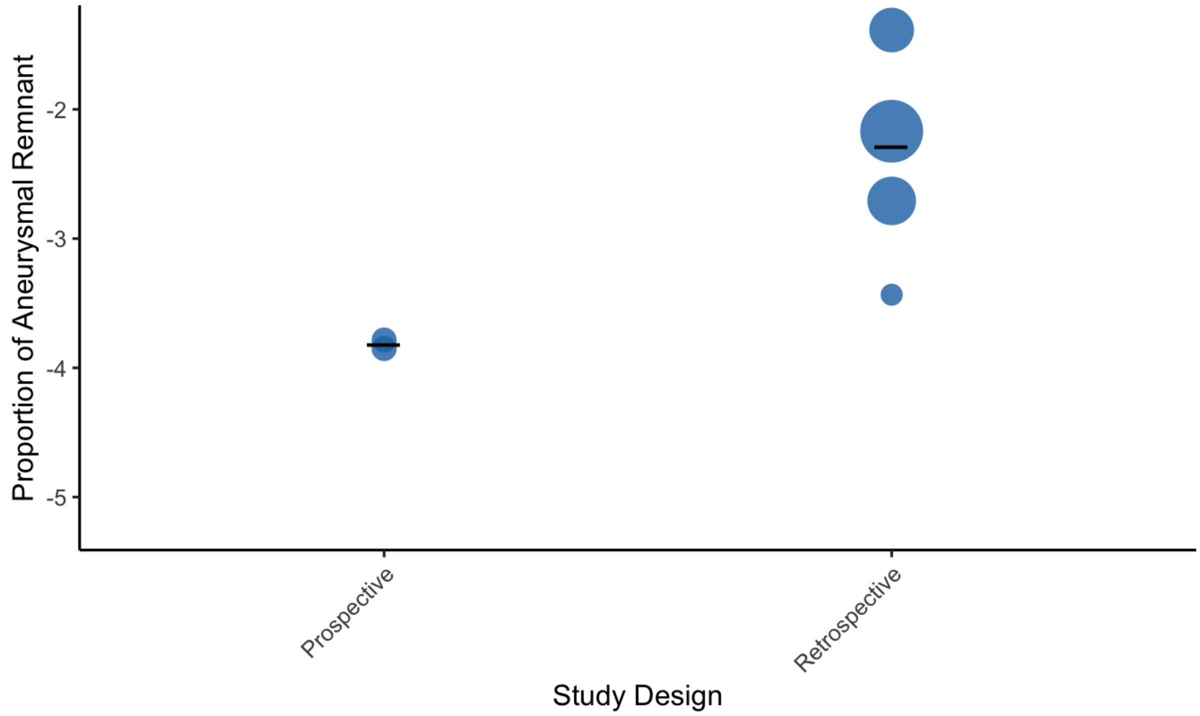

Supplementary Figure 13 – Meta-regression bubble plots: Study Design predicted Aneursym Remnant

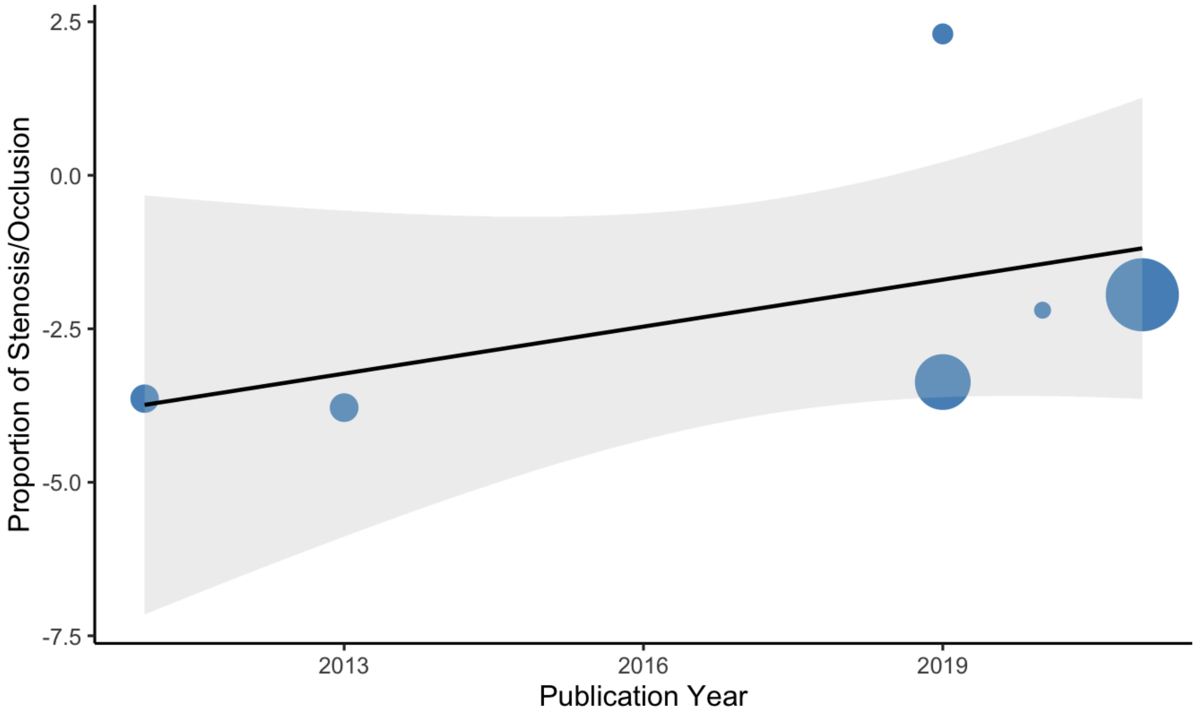

Supplementary Figure 14 – Meta-regression bubble plots: Publication Year predicted Stenosis/Occlusion

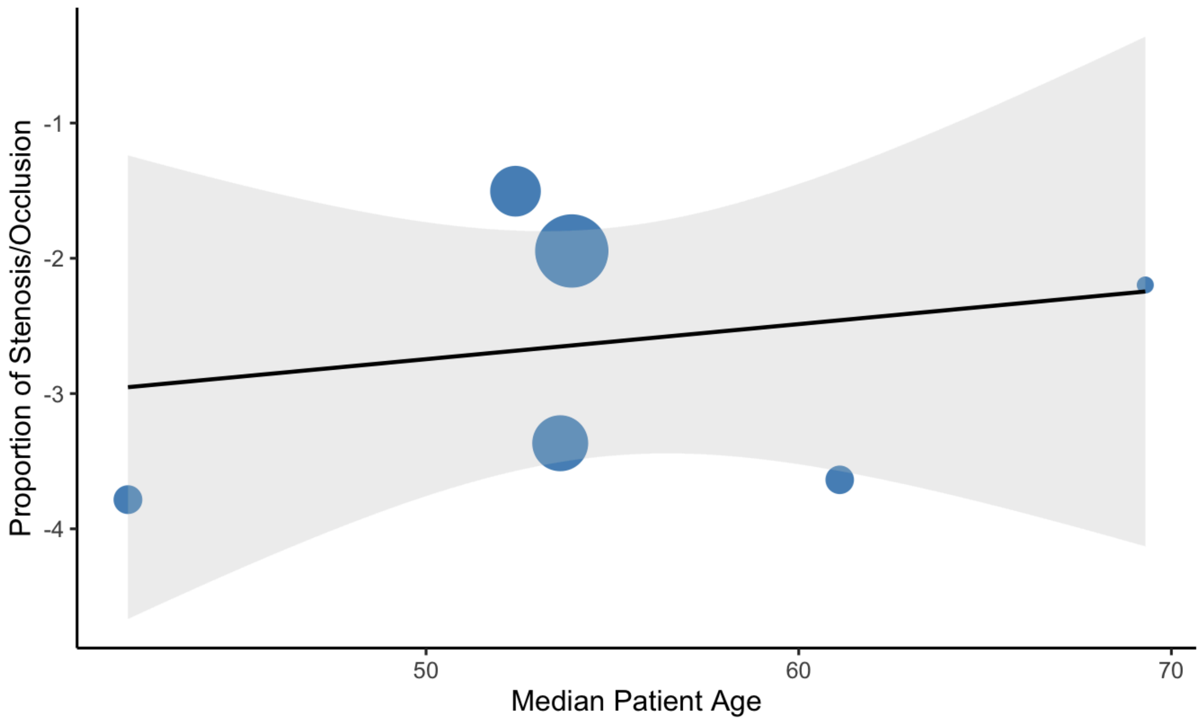

Supplementary Figure 15 – Meta-regression bubble plots: Median Patient Age predicted Stenosis/Occlusion

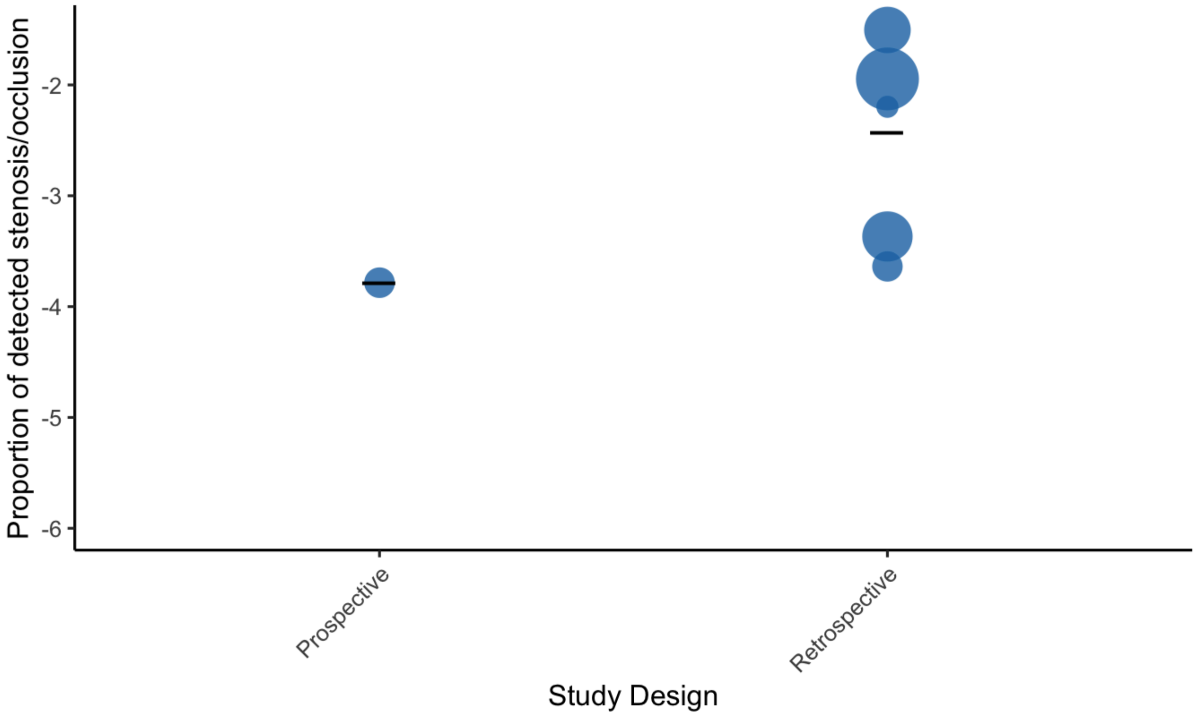

Supplementary Figure 16– Meta-regression bubble plots: Study Design predicted Stenosis/Occlusion

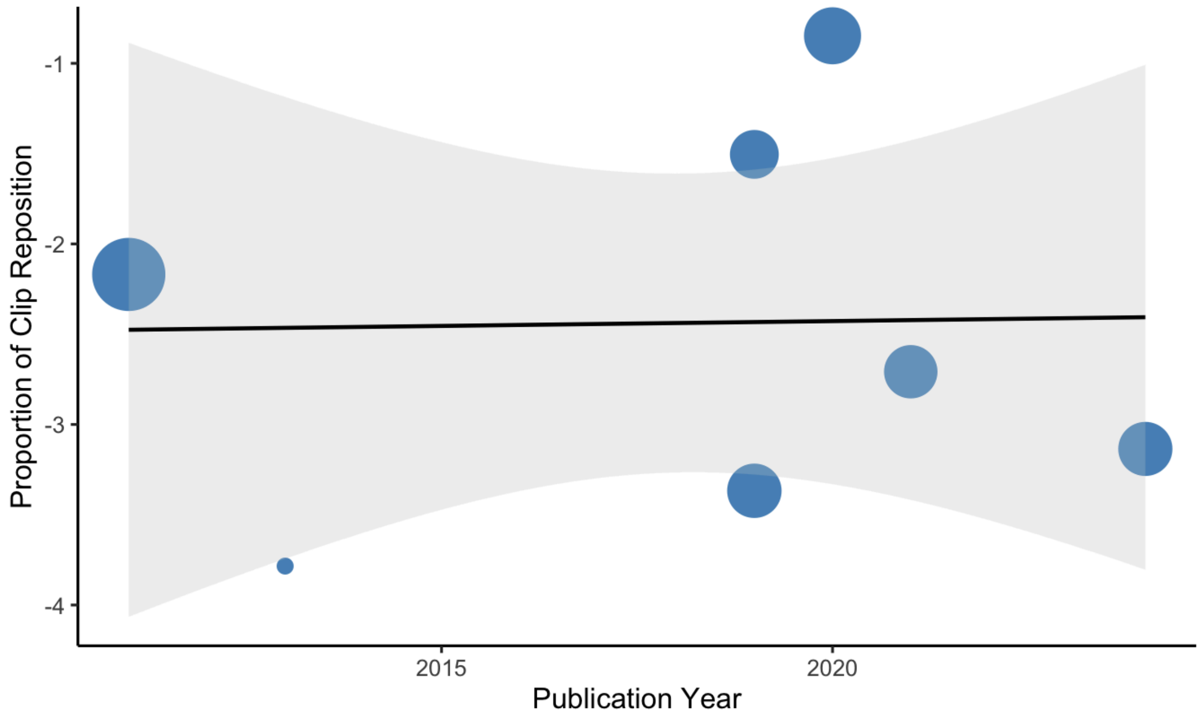

Supplementary Figure 17 – Meta-regression bubble plots: Publication Year predicted Clip Reposition

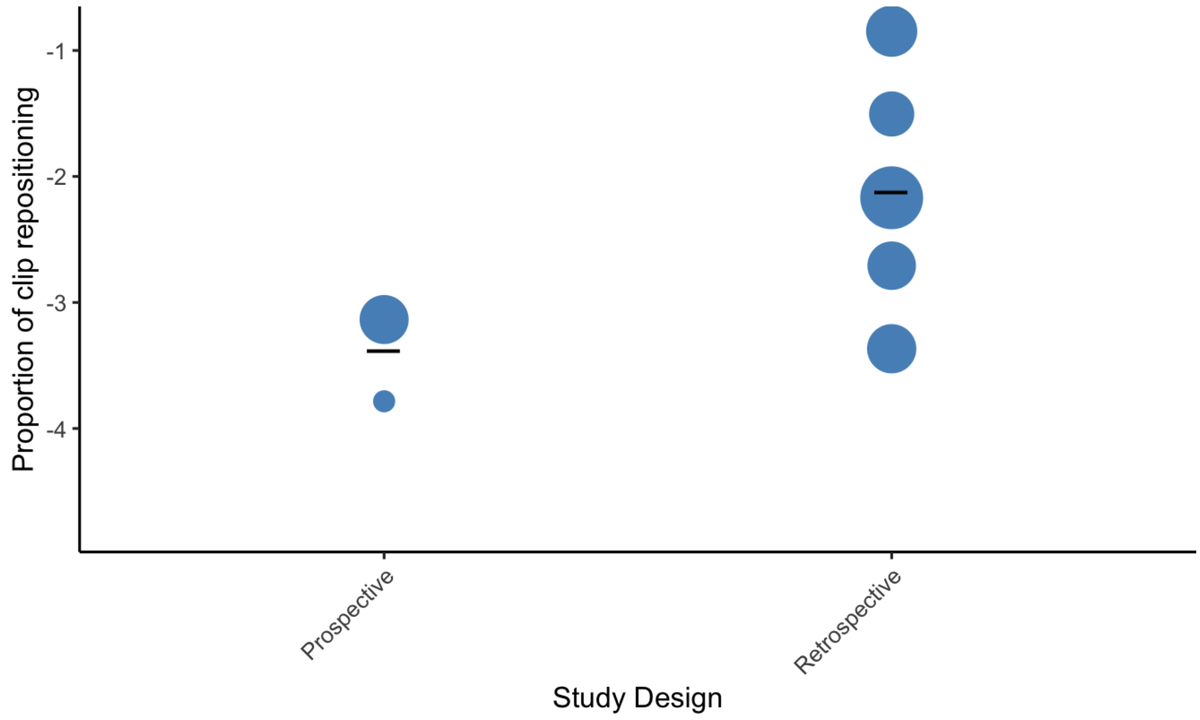

Supplementary Figure 18 – Meta-regression bubble plots: Study Design predicted Clip Repositioning

Supplementary Statistical Note

Meta-regression analyses were performed using the Freeman–Tukey double–arcsine transformed proportions to stabilize variance across studies.
